# Dysbiosis of skin microbiome and gut microbiome in melanoma progression

**DOI:** 10.1186/s12866-022-02458-5

**Published:** 2022-02-25

**Authors:** Chahrazed Mekadim, Helena Kupcova Skalnikova, Jana Cizkova, Veronika Cizkova, Anna Palanova, Vratislav Horak, Jakub Mrazek

**Affiliations:** 1 Laboratory of Anaerobic Microbiology, Institute of Animal Physiology and Genetics of the Czech Academy of Sciences, Videnska 1083, 142 20 Prague, Czech Republic; 2 Laboratory of Applied Proteome Analyses, Institute of Animal Physiology and Genetics of the Czech Academy of Sciences, Rumburska 89, 277 21 Libechov, Czech Republic; 3 Department of Radiobiology, Faculty of Military Health Sciences, University of Defence, Trebesska 1575, 500 01 Hradec Kralove, Czech Republic; 4 Department of Cell Biology, Faculty of Science, Charles University, Vinicna 7, 128 00 Prague, Czech Republic

**Keywords:** Melanoma, Skin cancer, Tumour microenvironment, MeLiM, Pig, Skin microbiome, Gut microbiome, Gut-skin axis, Dysbiosis, Metagenomic analysis, NGS

## Abstract

**Background:**

The microbiome alterations are associated with cancer growth and may influence the immune system and response to therapy. Particularly, the gut microbiome has been recently shown to modulate response to melanoma immunotherapy. However, the role of the skin microbiome has not been well explored in the skin tumour microenvironment and the link between the gut microbiome and skin microbiome has not been investigated in melanoma progression. Therefore, the aim of the present study was to examine associations between dysbiosis in the skin and gut microbiome and the melanoma growth using MeLiM porcine model of melanoma progression and spontaneous regression.

**Results:**

Parallel analysis of cutaneous microbiota and faecal microbiota of the same individuals was performed in 8 to 12 weeks old MeLiM piglets. The bacterial composition of samples was analysed by high throughput sequencing of the V4-V5 region of the 16S rRNA gene. A significant difference in microbiome diversity and richness between melanoma tissue and healthy skin and between the faecal microbiome of MeLiM piglets and control piglets were observed. Both Principal Coordinate Analysis and Non-metric multidimensional scaling revealed dissimilarities between different bacterial communities. Linear discriminant analysis effect size at the genus level determined different potential biomarkers in multiple bacterial communities. *Lactobacillus, Clostridium *sensu stricto 1 and *Corynebacterium* 1 were the most discriminately higher genera in the healthy skin microbiome, while *Fusobacterium, Trueperella, Staphylococcus, Streptococcus* and *Bacteroides* were discriminately abundant in melanoma tissue microbiome. *Bacteroides, Fusobacterium* and *Escherichia-Shigella* were associated with the faecal microbiota of MeLiM piglets. Potential functional pathways analysis based on the KEGG database indicated significant differences in the predicted profile metabolisms between the healthy skin microbiome and melanoma tissue microbiome. The faecal microbiome of MeLiM piglets was enriched by genes related to membrane transports pathways allowing for the increase of intestinal permeability and alteration of the intestinal mucosal barrier.

**Conclusion:**

The associations between melanoma progression and dysbiosis in the skin microbiome as well as dysbiosis in the gut microbiome were identified. Results provide promising information for further studies on the local skin and gut microbiome involvement in melanoma progression and may support the development of new therapeutic approaches.

**Supplementary Information:**

The online version contains supplementary material available at 10.1186/s12866-022-02458-5.

## Background

Cutaneous Melanoma (CM) is a malignant skin cancer originating from epidermal melanocytes [1, 2]. Even though it is less common than other skin cancers, CM is more lethal due to its high metastatic potential [2–4]. Considering the aggressiveness of this disease, it is important to identify the risk factors associated with melanoma development to improve diagnosis and treatment methods of this serious skin cancer. Different risk factors are associated with melanoma development: besides the genetic predisposition, such as the familial history of melanoma or other skin cancers and type of melanocytic nevi, other environmental factors, particularly the sun and UV radiation exposure, increase the risk of melanoma development [4–6].

Recently, the host microbiome is considered as a new component of the tumour microenvironment that influences tumour cell metabolism and plays a role in the cancer pathogenesis and treatment response [7, 8]. The commensal microbes interact directly with the cancerous cells of the tumour tissue, in which they are residing. Indirect effects could occur when the tumour development is affected by the metabolites of the microbiome from another location or by the administration of probiotics in the host diet [9].

Many observations suggested that the skin commensal microbiome may promote skin immunity and confer the host defence including the protection against skin inflammatory disorders, infections, wounds and skin cancer [10–19]. Several studies have suggested a potential role of the microbiota in skin carcinogenesis [20]. A reduced rate of skin cancer was observed in germ-free rats [21]. Similarly, different microorganism-associated molecular patterns (MAMPs) were identified as the trigger of receptors responsible for the inflammatory response that leads to tumour development, while skin inflammation in response to tumour promoters was reduced in mice lacking receptors [22–24].

Several studies have investigated the gut-skin connection in various skin diseases including skin cancer [25–27]. The gut-skin axis indicates that the gut microbiota and its metabolites can have a critical role in the development or prevention of skin cancers, including melanoma. In human, gut microbiota and its metabolites may have a mechanistic impact on antitumor immunity and immunotherapy in patients with advanced melanoma [28–37]. Also, it has been reported that the administration of selected strains from commensal intestinal microbiota may establish anti-tumour immunity and restrict melanoma growth in germ-free WT mice [38].

In order to investigate changes in the gut and skin microbiota composition during melanoma development, swine models are highly suitable due to the high similarities with human in terms of skin and gastrointestinal anatomy and physiology, genetics, immunology and pathophysiology of many human diseases [39–41]. The Melanoma-bearing Libechov Minipig (MeLiM) is a unique large animal model of hereditary melanoma [42]. In the MeLiM strain, the melanomas occur only in animals with pigmented skin, as the white pigs lack melanocytes in their skin. The MeLiM piglets are born with several nodular melanomas or the melanomas develop postnatally. The melanomas mostly invade into deep dermis and subcutaneous fat (corresponding to Clark level IV to V in human staging). At the age of 8 to 12 weeks, the spontaneous regression of melanomas starts to occur in the majority of animals, which is characterized by lymphocyte infiltration, and flattering and colour fading of tumours due to the replacement of the tumour by fibrous tissue [43–46]. However, in approximately 20% of piglets, the melanoma progression develops which is characterized by tumour growth and spread by metastases [47], mainly into the lymph nodes and lungs, cachexia and animal growth retardation. The MeLiM model enables to study the melanoma spontaneous development without any therapeutic interventions, which is not possible in human.

The aim of the present study was to assess the association between the diversity, composition and function of the skin and faecal microbiome and melanoma development and to compare the bacterial composition in such entities using high throughput sequencing. The samples were collected from multiple sites (inner melanoma tissue, melanoma surface, healthy skin and stool) of experimental piglets at different ages throughout melanoma progression and melanoma spontaneous regression (experimental scheme is presented in Fig. S1). Findings could contribute to the characterization of skin and gut microbiome composition and modification, as well as functional mechanisms following melanoma progression.

## Results

Skin and stool microbiome samples were collected from MeLiM piglets with melanoma progression (*n* = 10) and spontaneous regression (*n* = 10), as well as from crossbreds of MeLiM and white strain with black skin and several melanomas with regressive disease course (*n* = 4) and were compared to control MeLiM x white crossbreds with melanoma-free white skin (*n* = 10).

A total of 13,747,282 sequences were obtained from different samples. The mean sequence length was 275 bp. The metagenomic analysis of each microbiota (faecal and cutaneous) was performed separately.

### Diversity analysis

**Fig. 1:**
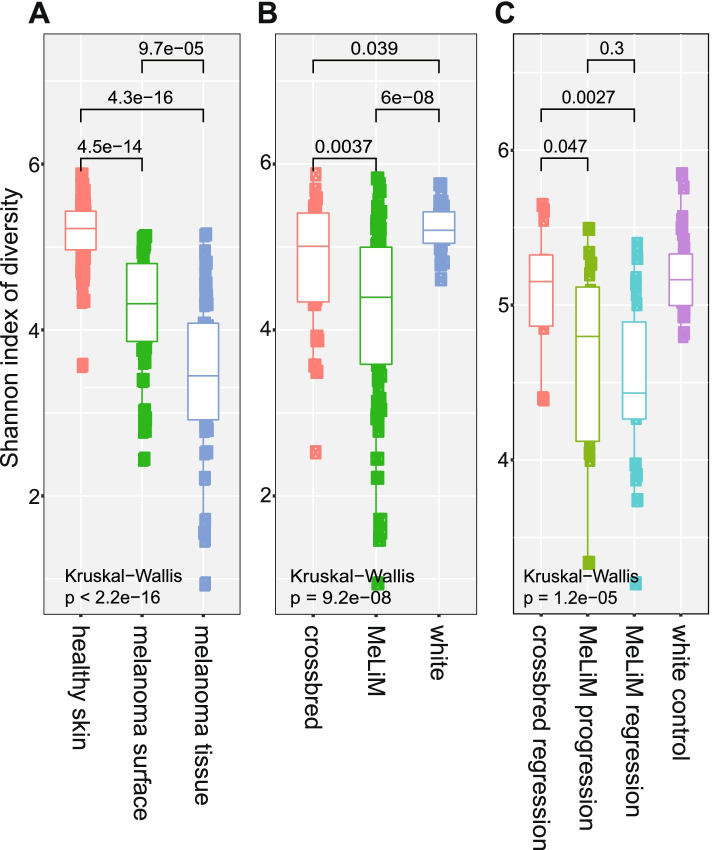
Box-plot of Shannon index of diversity **a**) in cutaneous microbiome among different cutaneous samples (healthy skin, melanoma surface and melanoma tissue), **b**) cutaneous microbiome of different animal breeds (white, crossbred, MeLiM) and **c**) faecal microbiome of different piglets groups (white control, crossbred with melanoma regression, MeLiM with melanoma regression and MeLiM with melanoma progression) using Kruskal–Wallis pairwise test (*p*-value ≤ 0.05) was used to compare between different samples

Alpha diversity of samples collected from different sites (melanoma tissue, melanoma surface, healthy skin and stool) was evaluated to determine the bacterial diversity of each animal phenotype (progression, spontaneous regression and white control) using Chao1, ACE, observed species, Fisher, Shannon, Simpson and Inverse Simpson indexes. The results are represented in the boxplot graph (Fig. 1, S2, S3). The obtained values after comparison between different groups are reported in (Table S1, S2). A highly significant difference in the skin microbiota diversity was markedly noticed between different sites of sampling (melanoma tissue, melanoma surface and healthy skin) and between different animal groups (MeLiM, white control, and black crossbred) (Kruskal–Wallis test; *p* < 0.05). The highest bacterial diversity was observed in the healthy skin microbiome of white control pigs (Fig. 1a, 1b, S2a, S2b). The diversity of melanoma surface and melanoma tissue in the MeLiM progressive group was lower than in the MeLiM with spontaneous regression but the differences were not significant (Fig. S3a). However, through the animal age, the diversity of the skin microbiome significantly increased in melanoma regression group while no distinct difference was observed in the skin microbiome of melanoma progression group between the analysed ages (Fig. S3b).

Similarly, in the faecal microbiota, the highest alpha diversity was observed in white control animals while the MeLiM animals with melanoma progression showed the lowest alpha diversity. (Fig. 1c, S2c). Also, no significant difference was observed between progressive and regressive melanoma groups, except a significant difference was noted between the faecal microbiota diversity of crossbred with melanoma regression and faecal microbiota diversity of MeLiM animals with melanoma regression (Fig. 1c, S2c). Throughout the age, the bacterial faecal community was dynamic and highly diverse in the white control animals and animals with melanoma regression (especially between ages 8 weeks and 10 weeks) whereas it was more stable in the animals with melanoma progression (Fig. S3c).

**Fig. 2:**
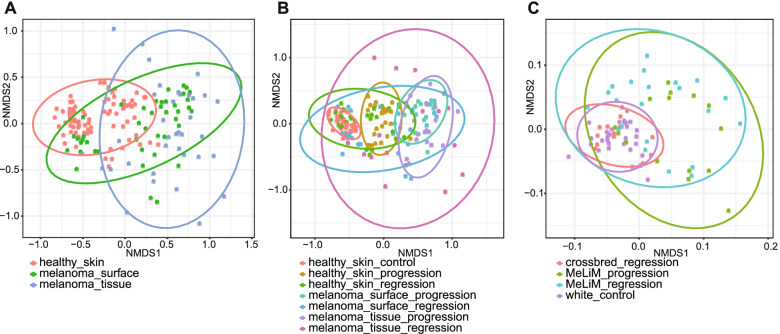
Non-metric Multidimensional Scaling (NMDS) plots based on the Bray Curtis distance matrix for beta diversity comparison of bacterial communities **a**) in skin microbiome among different samples (healthy skin, melanoma surface and melanoma tissue) **b**) in skin microbiome among multiple samples in different disease conditions (control, melanoma progression and melanoma regression) and **c**) in faecal microbiome of different piglets groups (white control, crossbred with melanoma regression, MeLiM with melanoma regression and MeLiM with melanoma progression). The confidence level of the ellipse was 95%

In addition, beta diversity was used to analyse the dissimilarities between bacterial communities in skin and stool samples separately. Principal Coordinate Analysis (PCoA) and Non-metric multidimensional scaling (NMDS) plots based on Bray Curtis distance were performed to reveal disparately separated microbial communities (Fig. 2, S4). In the skin microbiome, three major clusters were distinguished according to the nature of samples (melanoma tissue, melanoma surface and healthy skin) (Fig. 2a, S4a). Clusters of the progressive melanoma group and the control healthy skin group were distinctly separated showing the higher significant differences between those two bacterial communities (Fig. 2b, S4b). The cluster of regressive melanoma is the largest one which reflects the bacterial diversity dissimilarities between samples of this categorical group, which were heterogeneous because they belong to two different animal groups (MeLiM and Crossbred) and due to the changes in microbial composition throughout the ages. The bacterial diversity of faecal samples in crossbred with melanoma regression was more similar to that in white crossbred animals (control) and MeLiM progressive and regressive groups were clustered together indicating that no distinct difference was observed in faecal bacterial structures between the progressive and regressive melanoma (Fig. 2c, S4c).

### Relative abundance analysis and taxonomic composition

**Fig. 3:**
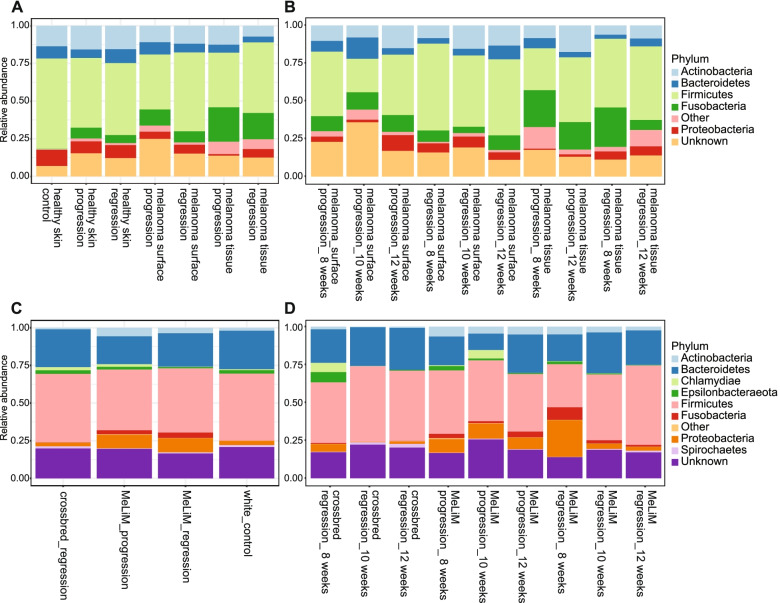
Relative abundance of the microbial population at the phylum level **a**) in skin microbiome among different cutaneous samples (healthy skin, melanoma surface and melanoma tissue) in healthy piglets or piglets with melanoma (control, melanoma progression and melanoma regression) **b**) in melanoma surface and melanoma tissue microbiome with melanoma regression and melanoma progression at different ages (8, 10, 12 weeks), **c**) in faecal microbiome of different piglets groups (white control, crossbred with melanoma regression, MeLiM with melanoma regression and MeLiM with melanoma progression) and **d**) in faecal microbiome of piglets with melanoma (crossbred with melanoma regression, MeLiM with melanoma regression and MeLiM with melanoma progression) at different (8, 10, 12 weeks)

The relative abundance composition of cutaneous skin microbiome at the phylum level showed that the higher abundance of *Firmicutes* and *Proteobacteria* were associated with skin devoid of melanoma and white control pigs, while the relative abundance of *Fusobacteria* was associated with melanoma tissue and melanoma surface in MeLiM piglets with melanoma progression and spontaneous regression (Fig. 3a), (Table S3). The *Firmicutes* and *Proteobacteria* ratios were lower in those last groups. *Actinobacteria* and *Bacteroidetes* abundances were relatively similar in all samples. The relative abundances of *Fusobacteria* were decreased dramatically in the regressive melanoma tissue microbiome from 20.9% at the age of 8 weeks to 5.0% at the age of 12 weeks, while no significant changes were observed in the bacterial composition of progressive melanoma skin microbiome throughout the ages (Fig. 3b), (Table S3).

The bacterial phylogenetic compositions of the faecal microbiota of piglets at the phylum level were dominated by *Firmicutes* and *Bacteroidetes*, but these were slightly less abundant in MeLiM piglets (Fig. 3c), (Table S4). The higher abundance of *Proteobacteria* and *Fusobacteria* were associated with faecal microbiota in MeLiM piglets (Fig. 3c). In faecal microbiota of MeLiM piglets with melanoma regression, the relative abundances of *Fusobacteria* and *Proteobacteria* dropped from 3.7%, and 18.7%, respectively, at the age of 8 weeks, to 1.3%, and 2.6%, respectively at the age of 12 weeks, while the relative abundances of *Firmicutes* and *Bacteroidetes* increased significantly from 28.4%, and 19.0%, respectively, at the age of 8 weeks, to the 40.4%, and 29.1%, respectively, at the age of 12 weeks. In MeLiM piglets with melanoma progression, no significant changes were observed in the bacterial composition of faecal microbiota throughout the monitored ages (Fig. 3d), (Table S4).

At the genus level, the microbiome of skin devoid of melanoma in white piglets was dominated by *Clostridium *sensu stricto 1, *Corynebacterium* 1, and *Lactobacillus* while the microbiome of melanoma tissue in the MeLiM piglets was dominated by *Fusobacterium, Staphylococcus, Trueperella* and *Streptococcus* (Table S3). The faecal microbiota of crossbred piglets was dominated by *Clostridium *sensu stricto 1*, Lactobacillus, Prevotella* 9*, Ruminococcus* and *Faeclibacterium.* The relative abundance of *Bacteroides* was significantly higher in the faecal microbiota of MeLiM piglets with melanoma progression (Table S4).

**Fig. 4:**
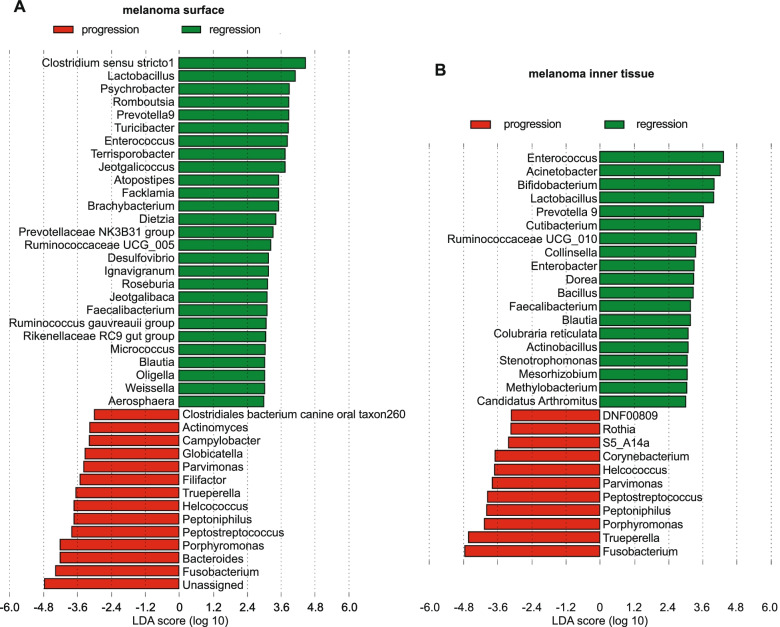
Linear discriminant analysis (LDA) effect size (LEfSe) at genera level (i) in skin microbiome between: **a**) melanoma surface in melanoma progression and melanoma regression and **b**) melanoma tissue in melanoma progression and melanoma regression. Differential abundance between categories was evaluated based on the factorial Kruskal–Wallis (KW) test and the pairwise Wilcoxon test (*p* < 0.05 and LDA score/effect-size threshold = 3)

The linear discriminant analysis (LDA) effect size (LEfSe) results (Fig. 4, S5) at genus level in skin microbiome showed that *Fusobacterium, Staphylococcus, Trueperella, Streptococcus, Peptostreptococcus* and *Peptoniphilus* were the more notable genera associated with melanoma microbiome and *Clostridium *sensu stricto 1*, Corynebacterium* 1*, Lactobacillus, Turicibacter, Terrisporobacter* and *Enterococcus* were bacterial genera most associated with healthy skin microbiome (Fig. S5a, S5b). Skin microbiome of piglets with progressive melanoma was related with *Fusobacterium, Trueperella, Bacteroides,* and *Porphyromonas* compared to the skin microbiome of piglets with regressive melanoma, where *Enterococcus, Acinetobacterium, Bifidobacterium, Lactobacillus* and *Prevotella 9* were discriminately abundant (Fig. 4a, b). In the faecal microbiome, LDA revealed a discriminant abundance of *Bacteroides* and *Fusobacterium* in MeLiM piglet with melanoma progression and *Escherichia-Shigella* in MeLiM piglets with melanoma regression (Fig. S5c). *Bacteroides, Escherichia-Shigella* and *Fusobacterium* were the most discriminant biomarkers in the faecal microbiome of MeLiM piglets while *Prevotella* 9*, Lactobacillus* and *Faecalibacterium* were the most discriminately abundant genera in the faecal microbiota of crossbred piglets (Fig. S5d).

These differences in relative abundances of certain genera reflect the dissimilarities in the skin and faecal microbiome between multiple experimental categories (melanoma tissue vs healthy skin, melanoma regression vs melanoma progression and MeLiM piglets vs crossbred piglets).

### Functional pathways analysis

To predict the potential function profiles of the skin and gut microbiota in piglets with melanoma progression or melanoma regression, the relative abundances of KEGG pathways were explored based on PICRUSt analysis. A total of 248 and 130 unique KEGG Orthology (KO) pathways at level 3 were predicted in the skin microbiome and faecal microbiome respectively (Table S5, S6).

At the second level of KEGG pathways, forty differently abundant pathways indicated a significant difference in the functions of the melanoma tissue microbiome compared with the healthy skin microbiome (Fig. S6a, S7a). Nineteen pathways were enriched in melanoma tissue microbiome, including Membrane transport, Translation, Glycan biosynthesis and metabolism, Nucleotide metabolism, Metabolism of cofactors and vitamins, Other ion-coupled transporters, Replication, Recombination and repairs of proteins, Infectious diseases, Protein folding and associated processing, Signalling molecules and interaction, Membrane and intracellular structural molecules, Amino acid metabolism, Translation proteins, Cell growth and death, Cell motility and secretion, Electron transfer carries, Digestive system and immune system diseases. There were eight most significant pathways associated with healthy skin microbiome including Amino acid metabolism, Xenobiotics biodegradation and metabolism, Carbohydrate metabolism, Sporulation, Lipid metabolism, Transcription, Metabolism of other amino acids, Energy metabolism in addition to pathways in cancers. Twenty-four KEGG pathways showed a significant difference in the potential function of melanoma tissue microbiome between melanoma regression and melanoma progression, fourteen of them were significantly enriched in progressive melanoma tissue microbiome, such as Replication and repair, Translation, Nucleotide metabolism, Metabolism of cofactors and vitamins, Metabolism of terpenoids and polyketides, Folding, sorting and degradation, Protein folding and association processing, Enzymes families. The relative abundance of Cell motility, Signal transduction, Sporulation, Biosynthesis and biodegradation of secondary metabolites pathways were significantly abundant in the regressive melanoma tissue microbiome (Fig. S6b, S7b).

In the faecal microbiome, twenty-eight KEGG pathways at level 2 revealed significant differences in potential functions between MeLiM and crossbred piglets (Fig. S6c, S7c). Membrane transport, Carbohydrate metabolism and Xenobiotics biodegradation and metabolism were the principal significant abundant functions associated with MeLiM faecal microbiota. Replication and repair, Translation, Cell motility, Amino acid metabolism, Nucleotide metabolism and Energy metabolism were the most significant pathways associated with the faecal microbiota of crossbred piglets. In the faecal microbiota of MeLiM piglets, five KEGG pathways at level 3 detected a significant difference in predicted functional profiles between regressive melanoma and progressive melanoma. Fatty acids biosynthesis and Lipid biosynthesis metabolism were significantly associated with progressive melanoma, while Selenocompound metabolism, Cell division (unclassified function) and General function prediction (unclassified) were the significant abundant pathways related to regressive melanoma (Fig. S6d, S7d). The results have shown that in addition to community differences, there may be differences in the functionalities of the microbiome between MeLiM piglets and healthy controls.

## Discussion

Several studies have reported the association of commensal microbiota of human or animal models with cancers, mostly focused on colorectal cancer [48–51], in addition to gastric [52, 53], liver [54], pancreatic [55, 56], lung [57–59], breast [60, 61] and bladder [62, 63] cancers. Generally, many reports suggested that microbiota induces carcinogenesis and other ones supported that microbiota play protective roles against cancer [64–67]. Recent reviews focused on the importance of the microbiome in skin cancer research and explored the crosstalk between the immune system and the skin microbiota in health and diseases (including cancer) [6, 11, 12, 68]. The profound reliance of the skin immune system on its resident microbiota for both host defence and tissue repair led to the integration of the skin microbiota and its metabolites as an intrinsic regulator of immune responses in the tissue microenvironments [13, 69–71]. The interactions between skin immune cells and microbiota are not only within the local microenvironment but also the skin immune system was stimulated by metabolites of microbes from other body sites (e.g. gastrointestinal tract) [9]. The gut microbiota is involved in cancer and is associated with anticancer therapy efficacy. Recently, several studies illustrated that the gut microbiota and the treatment with faecal microbiota transplantation (FMT) promoted the responses to anti-PD-1 immunotherapy and restored the tumour microenvironment in patients with advanced melanoma [28–30, 32, 34, 35, 72, 73].

In this study, the association between melanoma development and the changes in the bacterial composition of the gut and the skin microbiome were explored. The samples were collected from melanoma tissue, melanoma surface and healthy skin of porcine models: MeLiM piglets with melanoma progression or melanoma spontaneous regression and crossbred piglets with melanoma spontaneous regression or healthy (white) controls. Pigs are valuable large animal model due to similar anatomy and physiology, including metabolism and nutritional requirements to human. Human and pigs have been previously shown to share the major bacterial phyla (Firmicutes and Bacteroidetes) in both their gut and skin microbiome. Nonetheless, differences between human and pig have been found at the bacteria species level, which are expected to be caused mainly by the environmental factors, nutrition and age [74–76]. The pig breeding under uniform conditions enables to minimize the influence of environment and nutrition on the microbiome.

There are few studies about the link between skin cancer and skin microbiota in skin diseases [77–80]. The first work which studied the relationships between the human skin microbiome and melanoma has shown that the skin microbiome diversity was lower in patients with melanoma than in patients with melanocytic nevi. However, the authors did not detect the association between the cutaneous microbiome and melanoma [81]. Recently, the significant association of the skin microbiome in patients with acral melanoma was investigated at different stages [82]. In our previous study, we showed a significant difference between the healthy skin microbiome and melanoma tissue microbiome using DGGE method [83]. Similarly, in the present study, using high throughput sequencing of the 16Sr RNA gene, the metagenomic analysis revealed differences in skin microbiome of healthy skin, melanoma surface and melanoma tissue. Alpha diversity showed that the diversity in the healthy skin microbiome was significantly higher than in the melanoma microbiome. Also, the diversity of skin microbiome and faecal microbiome was significantly higher in crossbred white piglets (control animals without tumours) than in MeLiM piglets. The high diversity and richness of host microbiota are generally related to the host health stat [84–86]. Moreover, beta diversity based on Bray Curtis distance showed dissimilarities between microbial communities from multiple sites. The differences between regressive melanoma microbiome and progressive melanoma microbiome were not significant. However, a dynamic shift in the gut and cutaneous microbiome was explored in piglets with melanoma regression following the age, while the microbiota diversity was stable in MeLiM piglets with melanoma progression. That indicates that both the cutaneous microbiome and intestinal microbiome have a strong correlation with the melanoma process by age.

The dominant bacterial phyla in porcine skin microbiota were *Firmicutes, Actinobacteria, Proteobacteria* and *Bacteroidetes* in addition to *Fusobacteria,* which was abundant in the melanoma tissue microbiome of MeLiM piglets. The porcine faecal microbiota was mainly dominated by *Firmicutes* and *Bacteroidetes,* besides *Fusobacteria, Proteobacteria* and *Actinobacteria,* which were abundant in the faecal microbiota in MeLiM piglets. At the genus level, *Lactobacillus, Clostridium, Corynebacterium*1*, Terrisporobacter* and *Enterococcus* were associated with healthy skin of crossbred piglets whereas the relative abundance of *Fusobacterium, Staphylococcus, Trueperella, Streptococcus* and *Bacteroides* were discriminately higher in the melanoma microbiome. Consistent with our previous findings [86], *Fusobacterium necrophorum* subsp. *necrophorum* (18,3%) was the most abundant species in melanoma tissue microbiome of MeLiM piglets besides *Staphylococcus hyicus* (8,3%), *Trueperella pyogenes* (7,1%) *Streptococcus* (uncultured bacterium) (4,3%) and *Staphylococcus chromogenes* (2,7%), while they were considerably low or absents in the healthy skin microbiome. The relative abundance of *Lachnospiraceae* (9,7%), *Bacteroides* (4,2%), *Escherichia-Shigella* (2,4%) and *Fusobacterium* (1,6%) (*Fusobacterium necrophorum* subsp. *necrophorum* and *Fusobacterium gastrosuis*) were significantly discriminant bacterial genera in the faecal microbiota of MeLiM piglets, while *Prevotella* 9 (8,3%), *Prevotellaceae* NK3B31 group (4,4%), *Lactobacillus* (5,8%) and *Feacalibacterium* (1,9%) were the most discriminant bacterial genera in the faecal microbiota of crossbred piglets.

*Fusobacterium* was associated with pathogenesis in both human and livestock infections [87, 88]. Two subspecies of *F. necrophorum* are recognized. The subsp. *necrophorum* is more frequently present animal infections, while subsp*. funduliforme* was isolated from clinical human infections and their virulence is determined by secreting leukotoxin. In humans, *F. necrophorum* is responsible for Lemierre syndrome, which begins as bacterial pharyngitis and rapidly progresses to septic thrombophlebitis of the jugular vein [87–90]. *Fusobacterium nucleatum* was enriched in colorectal carcinoma. It was considered as a risk factor for the progression and severity of pancreatic and colorectal cancers [91, 92]. The active invader species *F. nucleatum* and *F. periodonticum* can independently invade host cells*. Fusobacterium nucleatum* colonized breast cancer tissue and colorectal cancer tissue, promoted tumour growth and caused cancer progression by inducing immunosuppression using extracellular adhesion and invasion molecule *Fusobacterium* adhesion (FadA) [93–98]. Gur et al. have demonstrated that the direct interaction of the Fap2 protein of *F. nucleatum* with the immune cells inhibitory receptor TIGIT (T cell immunoglobulin and ITIM domain) protected melanoma tumours bounded with *F. nucleatum* from NK cell cytotoxicity and T-cell activity [98]. Kalora et al. have identified 11 HLA-bound peptides derived from *F. nucleatum*, *Staphylococcus aureus* and *Staphylococcus capitis* inside melanoma tumour cells that elicited the immune response [99, 100].

*Staphylococcus hyicus* is the major causative agent of piglets’ exudative epidermitis [101]. *Staphylococcus chromogenes* has been identified as a frequent cause of bovine mastitis and intramammary infections [102, 103]. Interestingly, colonization of mice with *Staphylococcus epidermidis,* a skin commensal bacteria producing 6-N-hydroxyaminopurine (6-HAP), has reduced the incidence of ultraviolet-induced tumours [10]. *Trueperella pyogenes* causes diverse diseases in animals like mastitis, liver abscesses and pneumonia and it is rarely a cause of infection in humans [104–106].

*Lactobacillus* and *Corynebacterium* species have been shown to produce immunostimulatory metabolites leading to anti-cancer effects. *Lactobacillus johnsonii* was conducted to an immune-stimulatory effect by producing inosine which is a modulator of response to immune checkpoint blockade therapy and strongly enhanced the antitumor capacities of T cells in different tumour models including colorectal cancer, bladder cancer, and melanoma, by inducing Th1 differentiation through the inosine-A2AR-cAMP-PKA pathway [107]. A recent study showed that extracellular vesicles derived from *Lactobacillus rhamnosus* GG showed direct anti-tumour effects on hepatic cancer cell growth [108]. Also, the oral administration of lipoteichoic acid from *Lactobacillus rhamnosus* decreased the number of UV-induced skin tumours in SKH-1 hairless mice [109] and the administration of *Lactobacillus acidophilus* may reduce the incidence of colorectal cancer in rat models [110]. The antitumor effect of *Corynebacterium parvum* has been demonstrated since a very early time [111–113], and it was used as an immunostimulant and antitumor agent. Indeed, the intratumoral injection of *Corynebacterium granulosum* and *Corynebacterium parvum* in hamster melanoma showed regression of the tumour and reduction in the number of lung metastases [114, 115]. Lipton et al. indicated a decreased relapse rate and prolonged survival in patients in stage II, but not in stage I, of malignant melanoma treated with *C. parvum* when compared with BCG treated patients [116]. Nonetheless, no significant results were observed during the administration of *Corynebacterium parvum* followed by chemotherapy in patients with metastatic malignant melanoma compared with the group receiving chemotherapy alone [117]. Though, *Corynebacterium sp* was clinically associated with the progression of acral melanoma [82]. In our results, LDA detected two biomarkers genera from *Corynebacteriaceae* family: *Corynebacterium*1, which was discriminately abundant in healthy skin microbiome (Fig. S5a, S5b), (Table S3) and *Corynebacterium*, which was associated with melanoma progression (Fig. 4b), (Table S3).

Among the other bacteria affecting the immune response in cancer, *E. coli* produces colibactin, which may induce adenocarcinomas in human colorectal cancer patients [118]. *Bacteroides fragilis* led to promote colon tumorigenesis by overstimulating immune responses via T helper 17 (Th17) cells in mouse colorectal cancer model [119]. *Clostridium* species may suppress tumour growth in the liver and melanoma by restoring antitumour immunity [120, 121]. Gut microbiome enriched with *Faecalibacterium* was correlated with increased Immune Checkpoint Inhibitors response and improved immunotherapy response in mouse models and humans with metastatic melanoma [30, 122]. In a study of metastatic melanoma treated with anti-PD1 immune checkpoint blockade, the patients have reacted differently, responded and non-responded. The diversity of the faecal microbiome of the responders' patients was higher with increased abundance of the *Ruminococcaceae* and *Faecalibacterium,* while an increased abundance of *Bacteriodales* and a much lower bacterial diversity were observed in the non-responders’ microbiomes. In animal models, FMT of human gut microbiome enriched in *Faecalibacterium* to germ-free mice with melanoma showed reduced tumour growth and increased immune response in the tumour microenvironment [30]. The decrease of the relative abundances of opportunistic pathogens in skin microbiota and faecal microbiota of piglets with melanoma regression revealed the maturation of host microbiota following the age when the bacterial composition shifted from dysbiosis to the “healthy” balanced microbiota.

Functional prediction pathways results suggested the potential role of host microbiota in melanoma development. Numerous pieces of evidence have been demonstrated that metabolic disorders involved in carcinogenesis and can be a target to treat cancer [123, 124]. Sporulation and Bacterial motility proteins pathways were significantly higher in melanoma-free skin of piglets (Fig. S8). It was shown that Flagellin, the structural protein subunit of the bacterial flagellum, played a role as an adjuvant, immunomodulator, anti-tumour agent (in melanoma, colon, breast, lungs and prostate cancers) and radioprotective agent [125–127]. *Salmonella Typhimurium* flagellin stimulates NK cells to produce interferon-γ (IFN-γ) [128]. The flagellin genes (*fliC*) were detected in *Clostridium chauvoei, Clostridium haemolyticum*, *Clostridium novyi* types A and B, and *Clostridium septicum* [129]. Bacterial flagellin enhanced the antitumor response of the activated CD8 + T cells via TLR5 activation. Consequently, the perforin and granzyme proteins were secreted by activated CD8 + T cells and efficiently killed tumour cells [128]. Additionally, a significant reduction in tumour mass was observed after injection of flagellin into human colorectal tumours xenografted into nude mice [130]. Importantly, the vaccination of mice with syngeneic B16-OVA melanoma–derived plasma membrane vesicles engrafted with flagellin-related peptides 9Flg or 42Flg induced a dramatic inhibition of tumour growth and metastasis and resulted in complete tumour regression in lungs [131]. *Clostridium novyi*-NT (non-toxic) is a highly mobile spore-forming organism. Promising antitumour responses in both canine and human clinical studies were described after intratumoral injection of *Clostridium novyi*-NT spores [132]. Moreover, clostridial spores were considered as an ideal delivery vehicle for anti-cancer agents due to their selective germination in the hypoxic regions of solid tumour, their wide and fast dispersion throughout the tumour and their stability to oxygen and harsh conditions which allowed the immune system to recognize and destroy cancer cells efficiently [133].

Transporters, ABC transporters, Ribosome, Other ion-coupled transporters_Unclassified, Pyrimidine metabolism, Purine metabolism, Replication, Recombination and repair proteins, Aminoacyl-tRNA biosynthesis, Lipopolysaccharide biosynthesis proteins, Lipopolysaccharide biosynthesis, Bacterial secretion system and Ribosome biogenesis were highly predicted pathways affected by microbiota in MeLiM melanoma tissue (Fig. S8). Recently, ribosome synthesis was designated as a new target in cancer therapy. Moreover, recent research indicated the potential role of ribosomes compositions in tumorigenesis. The increase in ribosome biogenesis was noted in cancer cells which led to an elevation in protein synthesis and unrestrained growth [134]. The production of lipopolysaccharide (LPS) was potentially promoted by *Fusobacterium* which were abundant in melanoma tissue. LPS from *Fusobacterium* led to skin inflammation and Shwartzman reaction in rabbits [135]. LPS may promote inflammation via TLR4-mediated NF-kB activation and the production of different inflammatory factors, such as TNF-α, IL-6, and IL-1β [136, 137]. Many studies indicated the capacity of LPS to be involved in the progression of various cancers: breast cancer via a ‘MyD88-BLT2’-linked signalling cascade [138], prostate cancer by activating the NF-κB pathway [139], gastric cancer through the LPS-NF-κB-PD-L1 axis [140], and oesophageal cancer [141]. A recent study demonstrated the co-stimulation with *Trueperella pyogenes* pyolysin and LPSs induced autophagy and ATF6-dependent mechanism in endometrium stromal cells [142].

Amino acid metabolism is linked with tumour progression due to their indispensable role in cancer growth, cancer immunity and the tumour microenvironment [143, 144] and therapeutic means for targeting amino acid metabolism were suggested [145]. A potential antitumor effect of a combination of ascorbic acid, lysine, proline, arginine and green tea extract was investigated on human colon cancer cells HCT 116 in vivo (xenograft into male nude mice). Histological studies showed that the mixture supplementation strongly suppressed the growth of tumours by inhibiting Matrix metalloproteinases expression and invasion without toxic effects [146]. Tumour associated myeloid cells have the ability to suppress the protective anti-tumour immune response by targeting arginine metabolism and reducing arginine levels by producing arginases [147, 148]. In line with these findings result from our previous metabolomic study, where a highly significantly decreased level of arginine in plasma of MeLiM pigs with progression was the most striking difference compared to pigs with spontaneous regression [149]. Up-regulation of proline in melanoma cells compared to melanocytes was observed by de Ingeniis et al. [150]. An inhibition of the gene *ALDH18A1* encoding pyrroline-5-carboxylate synthase regulating proline biosynthesis in melanoma cells significantly decreased cultured melanoma cell viability and tumour growth [151, 152].

The predicted functional pathways affected by microbiota were significantly different between regressive melanoma microbiome and progressive melanoma microbiome. It is known that tumour cells accumulate several mutations and changes in metabolic pathways that might lead to cancer proliferation and metastasis. Ribosomes, DNA repair and recombination proteins, Pyrimidine metabolisms, Purine metabolisms were significantly higher in melanoma progression while Bacterial motility proteins. Flagellar assembly, Sporulation and Bacterial chemotaxis were significantly higher in melanoma spontaneous regression (Fig. S9). Consistent with our findings, many studies have assessed the interplay of pyrimidine metabolism in tumorigenesis [153, 154]. Comprehensively, the pyrimidine metabolic rate-limiting enzymes were highly expressed in lung, breast, colon, liver, stomach, and bladder cancer and played a key role in tumour cell proliferation [155]. Pyrimidine analogues acting as antimetabolites are used in cancer treatment for decades [156, 157]. Purines are basic components of nucleotides in cell proliferation, thus impaired purine metabolism is associated with the progression of cancer [158]. Notably, a high amount of purine metabolites has been noticed in tumour cells [159]. It was illustrated that *Escherichia coli* was able to target lung cancer cells using chemotaxis towards the biochemical factors secreted by carcinoma cells [160]. In faecal microbiota of MeLiM piglets, fatty acids biosynthesis and lipid biosynthesis metabolism were significantly associated with melanoma progression (Fig. S6d). Cancer cells as proliferative active cells require also lipids and fatty acids for cell growth, division, proliferation and survival. Deregulated lipid metabolism is an important metabolic phenotype of cancer cells [161]. Different mechanisms of fatty acids synthesis promoting tumour progression and metastasis were explored [162, 163]. Upregulations of several phosphatidylcholines were previously observed in MeLiM plasma of pigs with melanoma progression, compared to spontaneous regression [148]. A phospholipid derivative Lysophosphatidic acid (LPA) induces chemotaxis of melanoma cells and LPA degradation by melanoma cells forms a gradient in the tumour microenvironment that drove their spreading [164].

Ribosomes, DNA repair and recombination proteins and Pyrimidine metabolisms pathways, which were associated with melanoma progression in melanoma tissue microbiome, were significantly lower in the faecal microbiota of MeLiM piglets compared to crossbred piglets (Fig. S10) and they were significantly lower in the faecal microbiota of MeLiM piglets with melanoma regression at the age of 8 weeks compared to MeLiM piglets with melanoma regression at the age of 10 and 12 weeks (Fig. S11, S12). However, membrane transport pathways (Transporters, phosphotransferase system (PTS), ABC transporters, and others ion-coupled transporters unclassified) were significantly enriched in melanoma tissue microbiome, in the faecal microbiota of MeLiM piglets compared to crossbred piglets and they were significantly higher in the faecal microbiota of MeLiM piglets with melanoma regression at the age of 8 weeks (Fig. S8, S9, S10, S11, S12). Throughout the age, membrane transport pathways significantly decreased in the melanoma regression group while no significant difference was detected in the melanoma progression group in this pathway.

Alterations of membrane transport pathway were associated with several human diseases including cancer [141, 165, 166] and caused severe functional influences: increase intestinal permeability, alteration of the balance of substances between both sides of gut mucosa and destruction of the intestinal mucosal barrier. In addition, there is new evidence of the impact of the intestinal microbiome on skin physiology and microbiome [25, 167]. In case of disrupted intestinal barriers, intestinal bacteria and their metabolites have been reported to diffuse in the bloodstream, reach the skin, and disturb skin homeostasis [25, 27]. Based on those observations, our results lead up to deduce that in MeLiM piglets the intestinal permeability was higher and allowed the migration of the intestinal bacteria and its metabolites throughout the blood to gain access to the melanoma microenvironment and enhance tumour growth and proliferation. However, following the age, the intestinal permeability of piglets with melanoma regression was decreased and consequently, the transit of intestinal bacteria and its metabolites from the gut to the skin was restricted which was conducted to melanoma regression.

Depth studies suggested that gut *F. nucleatum* originated from the oral cavity [168, 169]. Several reports have demonstrated that *Fusobacteria* can migrate from the oral cavity to other districts of the body via blood circulation or lymphatic circulation systems to cause serious infections in various organs of humans including the head, neck, respiratory tract, brain, colon, liver and lymph nodes to cause metastatic abscess formation [170–175]. Also, recent studies suggested the translocation of *Fusobacteria* from colorectal cancer cells to metastatic sites attached with the primary tumour cells via Fap2, as part of metastatic tissue colonization [170, 175]. These observations indicate the importance of tumour microbiota as essential components of the tumour microenvironment.

## Conclusion

According to our knowledge, this is the first study exploring the association between gut and skin microbiome changes and melanoma progression. The implementation of MeLiM piglets as an animal model in melanoma progression might be a promising approach. Significant differences were observed in bacterial composition, relative abundances, richness and diversity indexes, and potential functional pathways in skin and gut microbiome of MeLiM piglets with melanoma progression compared to healthy piglets (controls). *Fusobacterium* and *Bacteroides* were the common potential biomarkers identified in both gut and skin microbiome in MeLiM piglets with melanoma progression.

The comprehensive analysis of the gut-skin axis is essential to understand the bidirectional cross-talk between the gut and skin tumour microbiome and is regarded as an exciting field of research, with promising therapeutic, dermatologic and cosmetic applications. Moreover, the evaluation of the functions of distinct bacteria (biomarker) and their metabolites in host physiology and cancer progression can provide novel insights into the underlying mechanisms and pathways to enhance the efficacy of both anticancer therapy and cancer prevention. Furthermore, a meta-analysis might be potentially expanded to include fungi and viruses in order to fully exploit the interaction network and potential functional between the gut and skin microbiome and melanoma development.

## Methods

### Animal experimentation

All animals used in the study are owned by the Institute of Animal Physiology and Genetics, Czech Academy of Sciences. The study was conducted under the Authorization for the use of experimental animals (No. 71922/2016-MZE-17214) and Authorization for breeding of experimental animals and delivery of experimental animals (No. 9322/2015 MZE-17214) issued by the Ministry of Agriculture of the Czech Republic and approved by the Resort Professional Commission of the Czech Academy of Sciences for Approval of Projects of Experiments on Animals (Projects of Experiments No. 82–2017 and No. 96–2015).

The MeLiM piglets (*n* = 20) and MeLiM x white control pig crossbreds (*n* = 14) were included in the study. The piglets were housed together with sows, fed by the diet appropriate to their age with unlimited access to water and sow milk. According to the phenotype, the piglets were divided into 4 groups: (i) MeLiM piglets with black skin and melanomas undergoing progression (*n* = 10); (ii) MeLiM piglets with black skin and melanomas undergoing spontaneous regression (*n* = 10); (iii) crossbreds with black skin and melanomas undergoing spontaneous regression (*n* = 4); and, (iv) crossbreds with white skin (controls without tumours; *n* = 10).

The melanoma development (the position, size and shape of tumours, as well as animal body weight) was macroscopically monitored at the 6, 8, 10 and 12 weeks of piglet age to assess the progressive or regressive disease development. At the 8, 10, and 12 weeks of age, the scrapings from the melanoma surface and surrounding healthy skin surface (4 cm^2^ area, approximately 5 cm from melanoma) were collected using a sterile scalpel blade to a sterile tube. Before the skin surface scraping, the bristles were removed by a different sterile scalpel blade. In these time intervals, the piglet faeces were collected from the rectum into sterile tubes. The scrapings and stool samples were immediately frozen to -80 °C. In addition, at 8 and 12 weeks of piglet age, the melanomas were collected from animals under Isoflurane and nitric oxide general anaesthesia. After collection, the tumour edges and surface were aseptically removed and the melanoma inner tissue was immediately frozen to liquid nitrogen and stored at -80 °C until analysis.

### DNA extraction

The DNA was extracted from stool samples using QIAamp PowerFecal DNA Kit (QIAGEN, Hilden, Germany) and from tissue samples using DNeasy PowerBiofilm Kit (QIAGEN, Hilden, Germany) as per the manufacturer’s protocols. The disintegration step was performed with a FastPrep-24 Classic instrument (MP Biologicals) device for 1 min at a maximum speed of 6.5 m/s. The elution was done with 60 μL of elution buffer. The eluted DNA was stored at − 20 °C until further use.

### Amplification of 16S rDNA and purification

The V4-V5 region of the 16S rRNA gene was amplified to prepare amplicons from the extracted DNA using the primer pair: BactB-F (GGATTAGATACCCTGGTAGT) and BactB-R (CACGACACGAGCTGACG) [176]. mixed with EliZyme HS FAST MIX Red (Elisabeth Pharmacon, Brno, Czech Republic). The PCR conditions were: denaturation for 5 min at 95 °C, followed by 25 cycles of 30 s at 95 °C, 30 s at 57 °C and 30 s at 72 °C, ending by final elongation for 5 min at 72 °C. The quality of PCR amplicons was checked by 1.5% agarose gel electrophoresis (30 min at 100 V), then the amplicons were purified using QIAquick PCR Purification Kit (QIAGEN, Hilden, Germany) according to the manufacturer’s protocol and the concentration of the purified amplicons was determined by Nanodrop OneC Microvolume UV–Vis Spectrophotometer (Thermo Scientific, Wilmington, USA).

### Semi-conductor based Next Generation Sequencing

For diversity analyses, libraries were prepared from purified amplicons of V4-V5 region of the 16Sr RNA gene (300 bp) by NEBNext®Fast DNA Library Prep Set kit (New England Biolabs, Ipswich, MA, USA). The adaptor-ligated libraries were purified using AMPure XP beads sizing (Beckman Coulter, Brea, CA, USA). The quality of purified libraries was controlled by High Sensitivity DNA electrophoresis with Agilent 2100 Bioanalyzer instrument (Agilent Technologies, Santa Clara, CA, USA) using the Agilent High Sensitivity DNA Reagents and chips (Agilent Technologies, Santa Clara, CA, USA). The purified libraries were quantified using the KAPA Library Quantification Kit for Ion Torrent Platforms (Roche, Pleasanton, CA, USA) in QuantStudio™ 3 Real-Time PCR System (Thermo Fisher Scientific, Waltham, MA, USA). The pool of equimolar concentration of barcoded libraries was used to prepare a sequencing template with Ion Sphere Particles (ISPs) using Ion PGM™ Hi-Q™ View OT2 400 Kit (Thermo Fisher Scientific, Waltham, MA, USA) in Ion OneTouchTM 2 instrument. The enrichment of the template positive ISPs was performed on the Ion OneTouchTM ES instrument. The enriched template positive ISPs were loaded in Ion 316TM Chip v2 BC (Thermo Fisher Scientific, Waltham, MA, USA). The sequencing was then performed on an Ion Torrent PGM sequencer (Thermo Fisher Scientific, Waltham, MA, USA) using Ion PGM™ Hi-Q™ View Sequencing solutions kit (Thermo Fisher Scientific, Waltham, MA, USA) following the manufacturer’s instructions.

### Microbiome analysis and statistical analysis

Bacterial 16S rRNA gene sequences were obtained in FASTQ format and analyzed by QIIME 2 version 2020.2 pipeline [177]. Quality filtering and chimaera excluding were performed using the DADA2 plugin in QIIME2 [178] (via q2‐dada2) to extract sequence variants (ASVs). Mafft was used to align the sequences [179] (via q2‐alignment) and fasttree was used to construct a phylogenetic tree [180] (via q2‐phylogeny). Then, clustering and taxonomy classification was generated using the qiime feature-classifier with VSEARCH based on SILVA database (version 132) with 99% threshold [181]. The rarefaction was conducted to normalize the data based on reads depth in all samples. Alpha diversity indexes were determined using q2‐diversity plugin based on the Kruskal–Wallis test and visualized using the qiime2R (https://github.com/jbisanz/qiime2R) and ggplot2 packages in R-Studio (version 3.6.3) [182–184]. Principal Coordinate Analysis (PCoA) based on Bray Curtis distance diversity metrics (beta diversity) were generated by qiime2 core-metrics phylogenetic pipeline after rarefaction. The 2-dimensional PCoA plots were generated by qiime2R and ggplot2. Non-metric multidimensional scaling (NMDS) plot was performed using phyloseq and ggplot2 and the dissimilarity was based on Bray Curtis distance [184, 185]. The confidence ellipse represents 95% of the confidence level. The linear discriminant analysis (LDA) with effect size (LefSe) algorithm [186] in Galaxy module http://huttenhower.sph.harvard.edu/galaxy/ was used to detect features with significant differential abundance between different biological categories of samples based on the factorial Kruskal–Wallis test and the pairwise Wilcoxon test to identify taxa with significant differential relative abundances at genus level with alpha values of 0.05 and a threshold value of 3.0 on the logarithmic LDA scores for discriminative features (*p* < 0.05 and LDA score/effect-size threshold = 3). Phylogenetic investigation of communities by reconstruction of unobserved states algorithm (PICRUSt v 2.3.0-b) [187] was applied to compare the potential function capacities of the cutaneous microbiome and faecal microbiome among different categories of samples. The functional genes were categorized into KEGG pathways at different subclasses levels 1, 2 and 3 and the resulting abundance table was imported in STAMP v2.1.3 program (Statistical Analysis of Metagenomic Profiles) for statistical analysis [188] by using Non-corrected Welch's t-test type two-sided, with the confidence interval (CI) method of Welch's inverted adjustment of 0.95 (*p* < 0.05). The relationships among functional capacities were analysed by principal component analysis (PCA).

## Supplementary Information

**Additional file 1. **Adiagram summarizing the principal steps of the used methods. Drawn by the authors using Adobe^Ò^Illustrator^Ò^CS6.

**Additional file 2. **Comparison of the alphadiversity of bacterial communities a) in cutaneous microbiome among differentcutaneous samples (healthy skin, melanoma surface and melanoma tissue), b)cutaneous microbiome of different animal breeds (white, crossbred, MeLiM) and c)faecal microbiome of different piglets groups (white control, crossbred withmelanoma regression, MeLiM with melanoma regression and MeLiM with melanomaprogression). Bacterial diversity and richness were estimated by differentalpha diversity indexes: Observed species, Chao, Ace, Shannon, Simpson, InverseSimpson and Fisher index. Kruskal–Wallis pairwise test (*p*-value ≤ 0.05) wasused to compare between different samples.

**Additional file 3. **Comparison of the bacterialalpha diversity a) in the cutaneous microbiome of piglets with melanomaregression and piglets with melanoma progression in different cutaneous samples(healthy skin, melanoma surface and melanoma tissue), b) in the cutaneousmicrobiome of piglets with melanoma progression and melanoma regression atdifferent ages (8, 10, 12 weeks) and c) in faecal microbiome of piglets atdifferent stat (control, melanoma progression and melanoma regression)throughout the age (8, 10, 12 weeks). Bacterial diversity and richness wereestimated by different alpha diversity indexes: Observed species, Chao, Ace,Shannon, Simpson, Inverse Simpson and Fisher index. Kruskal–Wallis pairwisetest (*p*-value ≤ 0.05) was used to compare between different samples.

**Additional file 4. **Beta diversity of bacterialcommunities using Principal Coordinate Analysis (PCoA) ordinations based on theBray Curtis distance matrix. The dissimilarities between bacterial communitieswere represented by regrouping in distinct clusters: a) in skin microbiomeamong different samples (healthy skin, melanoma surface and melanoma tissue, b)in skin microbiome among multiple samples in different disease conditions(control, melanoma progression and melanoma regression) and c) in faecalmicrobiome of different piglets groups (white control, crossbred with melanomaregression, MeLiM with melanoma regression and MeLiM with melanomaprogression). The confidence level of the ellipse was 95%.

**Additional file 5. **Linear discriminantanalysis (LDA) effect size (LEfSe) at genera level (i) in skin microbiomebetween: a) healthy skin and melanoma surface and b) healthy skin and melanomatissue, and (ii) in faecal microbiome c) between different conditions (healthycontrol, melanoma progression and melanoma regression) and d) between MeLiMpiglets and crossbred animals. Differential abundance between categories wasevaluated based on the factorial Kruskal-Wallis (KW) test and the pairwiseWilcoxon test (*p* < 0.05 and LDA score/effect-size threshold = 3).

**Additional file 6. **

**Additional file 7. **

**Additional file 8. **

**Additional file 9. **Functional pathway analysis ofthe cutaneous microbiome and faecal microbiome based on the KEGG database.Extended error bar plot identifying the significant differences in meanproportion (%) of predicted functional categories a) at second-level KEGGpathway between the healthy skin microbiome and melanoma tissue microbiome, b)at second-level KEGG pathway between melanoma progression and melanomaregression in melanoma tissue microbiome, c) at second-level KEGG pathwaybetween the faecal microbiome of MeLiM piglets and faecal microbiome ofcrossbred piglets and d) at third-level KEGG pathway between the faecalmicrobiome of MeLiM piglets with melanoma progression and MeLiM piglets withmelanoma regression using the STAMP software. The corrected *p*-values that wereshown on the right, were obtained from a Welch's t-test with the confidenceinterval (CI) method of Welch's inverted adjustment of 0.95 (*p*< 0.05).

**Additional file 10. **

**Additional file 11. **

**Additional file 12. **

**Additional file 13. **Principal ComponentAnalysis (PCA) of the predicted functional pathways at KEGG level 2 a) betweenthe healthy skin microbiome and melanoma tissue microbiome, b) between melanomaprogression and melanoma regression in melanoma tissue microbiome, c) betweenthe faecal microbiome of MeLiM piglets and faecal microbiome of crossbredpiglets and d) between the faecal microbiome of MeLiM piglets with melanomaprogression and MeLiM piglets with melanoma regression using the STAMP softwarebased on Non-corrected Welch'st-test type two-sided, with the confidenceinterval (CI)methodofWelch's invertedadjustment of 0.95(*p*< 0.05).

**Additional file 14. **

**Additional file 15. **

**Additional file 16. **

**Additional file 17. **Functional pathway analysis of cutaneous microbiomebased on the KEGG database. Extended error bar plot identifying the significantdifferences in mean proportion (%) of predicted functional categories at level3 of KEGG pathway between the healthy skin microbiome and melanoma tissue microbiome.

**Additional file 18. **Functional pathway analysisof cutaneous microbiome based on the KEGG database. Extended error bar plotidentifying the significant differences in mean proportion (%) of predictedfunctional categories at level 3 of KEGG pathway between melanoma progressionand melanoma regression in melanoma tissue microbiome.

**Additional file 19. **Functional pathway analysisof faecal microbiome based on the KEGG database. Extended error bar plotidentifying the significant differences in mean proportion (%) of predictedfunctional categories at level 3 of KEGG pathway between MeLiM piglets andcrossbred piglets.

**Additional file 20. **Functional pathway analysisof faecal microbiome based on the KEGG database. Extended error bar plotidentifying the significant differences in mean proportion (%) of predictedfunctional categories at second-level KEGG pathway between MeliM piglets withmelanoma regression at the age of 8 weeks and MeliM piglets with melanomaregression at the age of 10 weeks.

**Additional file 21. **Functional pathway analysisof faecal microbiome based on the KEGG database. Extended error bar plotidentifying the significant differences in mean proportion (%) of predictedfunctional categories at second-level KEGG pathway between MeliM piglets withmelanoma regression at the age of 8 weeks and MeliM piglets with melanomaregression at the age of 12 weeks.

**Additional file 22. **Pairwise comparison ofalpha diversity of cutaneous bacterial communities of animal models (white,crossbred, MeLiM piglets) in different skin samples (healthy skin, melanoma surfaceand melanoma tissue), at different stat (control, melanoma regression andmelanoma regression) at different ages (8, 10, 12 weeks) measured by Chao,Evenness, Shannon and Simpson index of diversity using Kruskal–Wallis pairwisetest (*p*-value ≤ 0.05).

**Additional file 23. **Pairwise comparison ofalpha diversity of faecal microbiota of animal models (white, crossbred, MeLiMpiglets) at different stat (control, melanoma regression and melanomaregression) at different ages (8, 10, 12 weeks) measured by Chao, Evenness,Shannon and Simpson index of diversity using Kruskal–Wallis pairwise test(*p*-value ≤ 0.05).

**Additional file 24. **The relative abundances ofthe bacterial community in skin microbiota of several cutaneous samples fromdifferent animal models at different ages at phylum and genus levels.

**Additional file 25. **The relative abundances ofthe bacterial community in the faecal microbiota of different animal models atdifferent ages at phylum and genus levels.

**Additional file 26. **Predicted functional KEGGpathways at levels 1, 2 and 3 in the skin microbiome of several cutaneoussamples (healthy skin, melanoma surface and melanoma tissue) from differentanimal models (white, crossbred, MeLiM piglets) at different disease states(control, melanoma regression and melanoma regression).

**Additional file 27. **Predicted functional KEGGpathways at levels 1, 2 and 3 in the faecal microbiota of different animalmodels (white, crossbred, MeLiM piglets) at different disease stat (control,melanoma regression and melanoma regression)

## Data Availability

The datasets used and/or analyzed during the current study are available from the corresponding author on reasonable request.

## Abbreviations

DGGE: Denaturing Gradient Gel Electrophoresis
FASTQ: Fast Adaptive Shrinkage Threshold Algorithm Quality
FMT: Faecal Microbiota Transplantation
KEGG: Kyoto Encyclopedia of Genes and Genomes
LDA: Linear Discriminant Analysis
LPS: Lipopolysaccharide
MeLiM: Melanoma-bearing Libechov Minipig
NMDS: Non-metric Multidimensional Scaling
PCA: Principal Component Analysis
PCoA: Principal Coordinate Analysis
PGM: Personal Genome Machine
PICRUST: Phylogenetic Investigation of Communities by Reconstruction of Unobserved States
STAMP: Statistical Analysis of Metagenomic Profiles
