# Supplementary figures and images for: Dysbiosis of skin microbiome and gut microbiome in melanoma progression
Source: BMC Microbiol. 2022 Feb 25;22:63. doi: 10.1186/s12866-022-02458-5 (PMC8881828; doi:10.1186/s12866-022-02458-5)

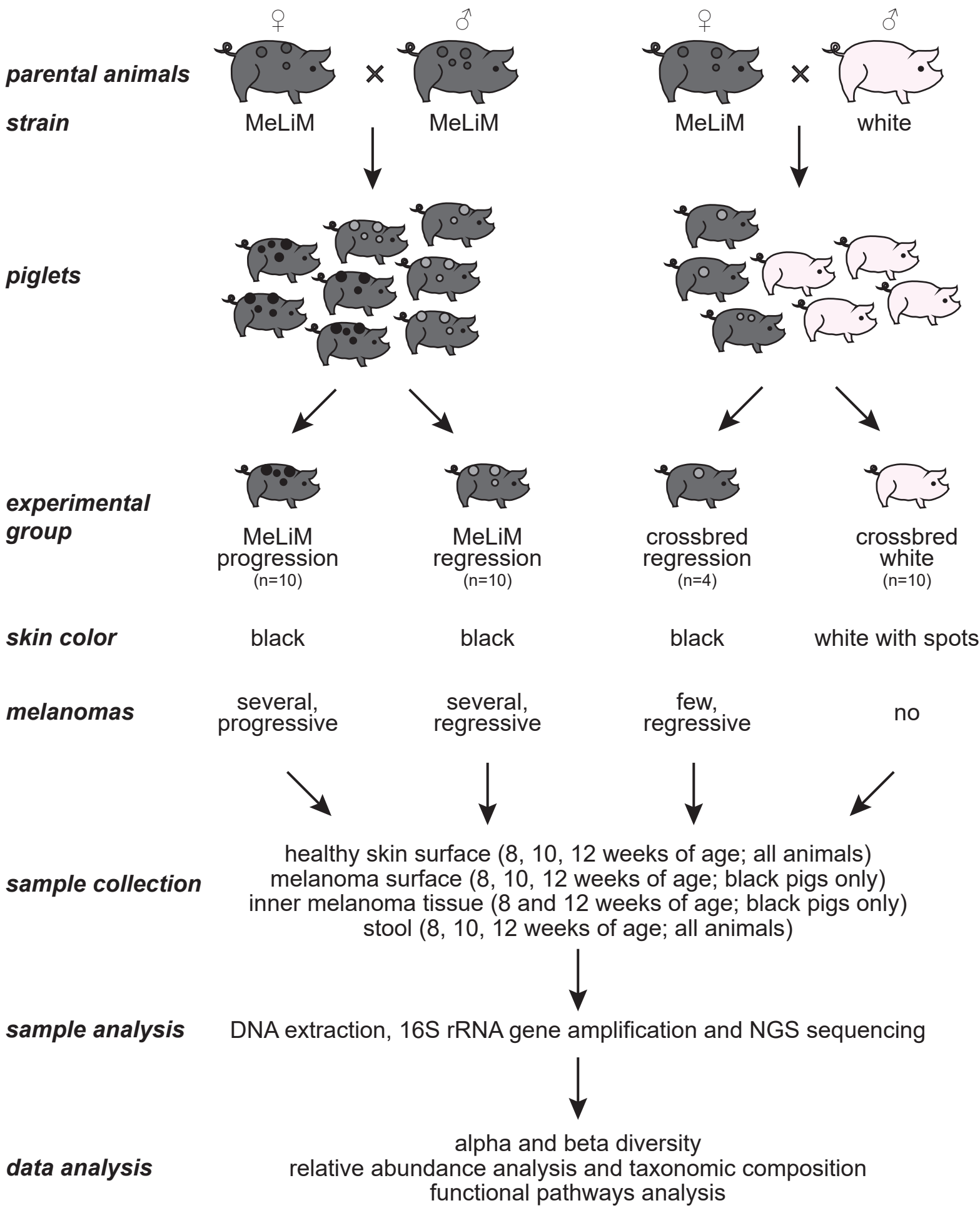

Supplement: Supplementary file 1 — Additional file 1. Adiagram summarizing the principal steps of the used methods. Drawn by the authors using AdobeÒIllustratorÒCS6. [file 12866_2022_2458_MOESM1_ESM.pdf]

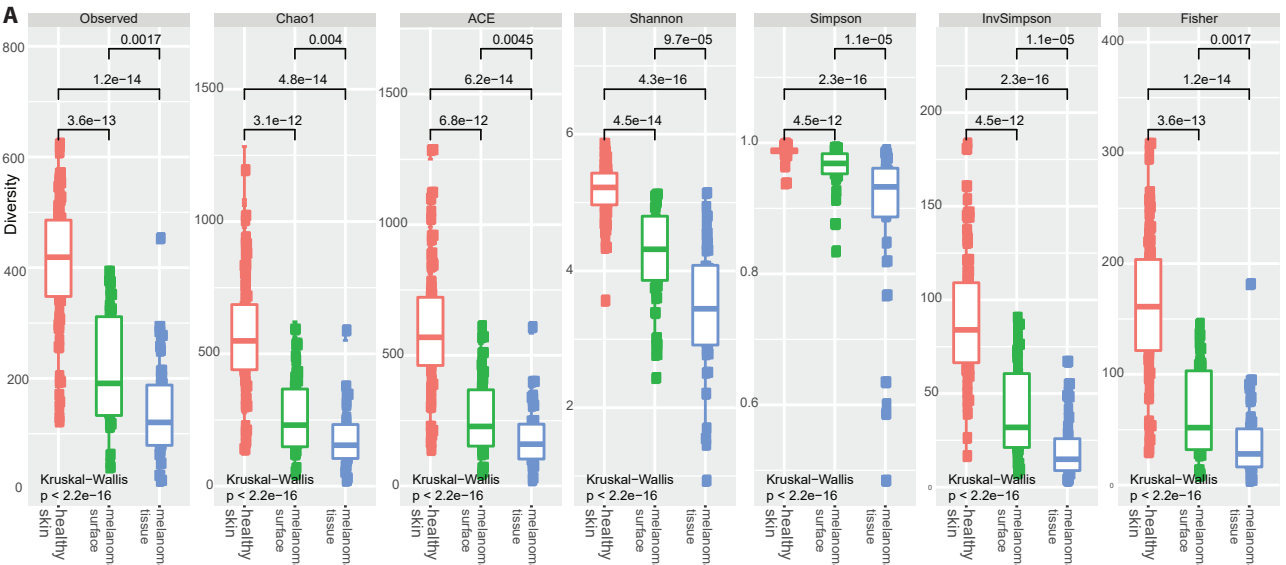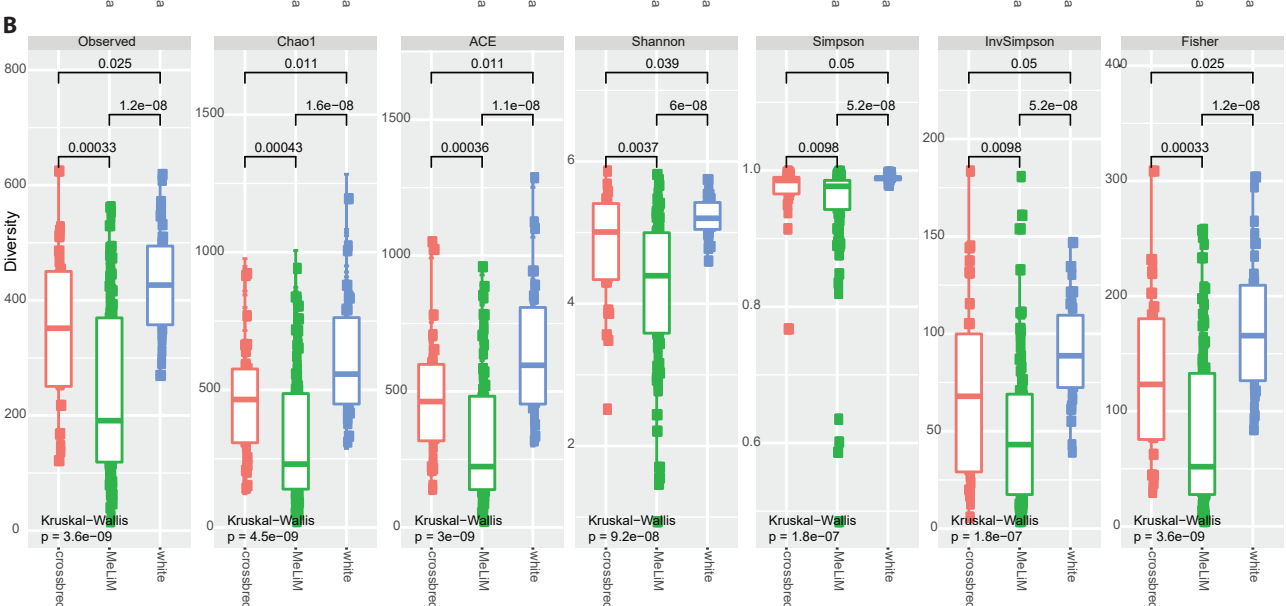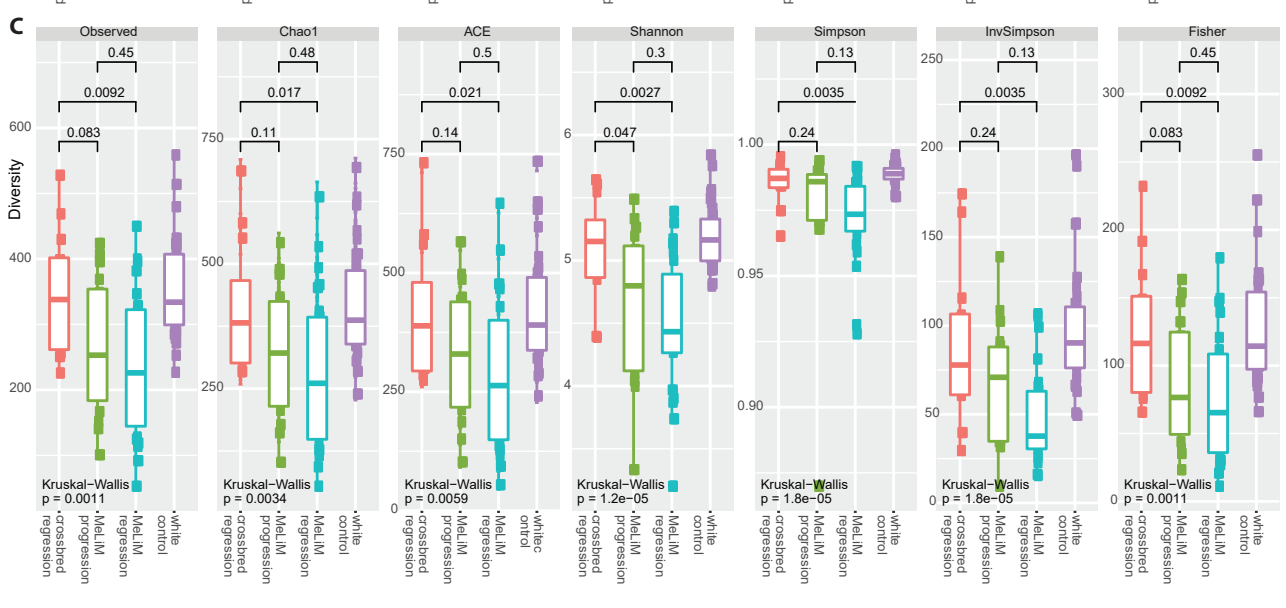

Supplement: Supplementary file 2 — Additional file 2. Comparison of the alphadiversity of bacterial communities a) in cutaneous microbiome among differentcutaneous samples (healthy skin, melanoma surface and melanoma tissue), b)cutaneous microbiome of different animal breeds (white, crossbred, MeLiM) and c)faecal microbiome of different piglets groups (white control, crossbred withmelanoma regression, MeLiM with melanoma regression and MeLiM with melanomaprogression). Bacterial diversity and richness were estimated by differentalpha diversity indexes: Observed species, Chao, Ace, Shannon, Simpson, InverseSimpson and Fisher index. Kruskal–Wallis pairwise test (p-value ≤ 0.05) wasused to compare between different samples. [file 12866_2022_2458_MOESM2_ESM.pdf]

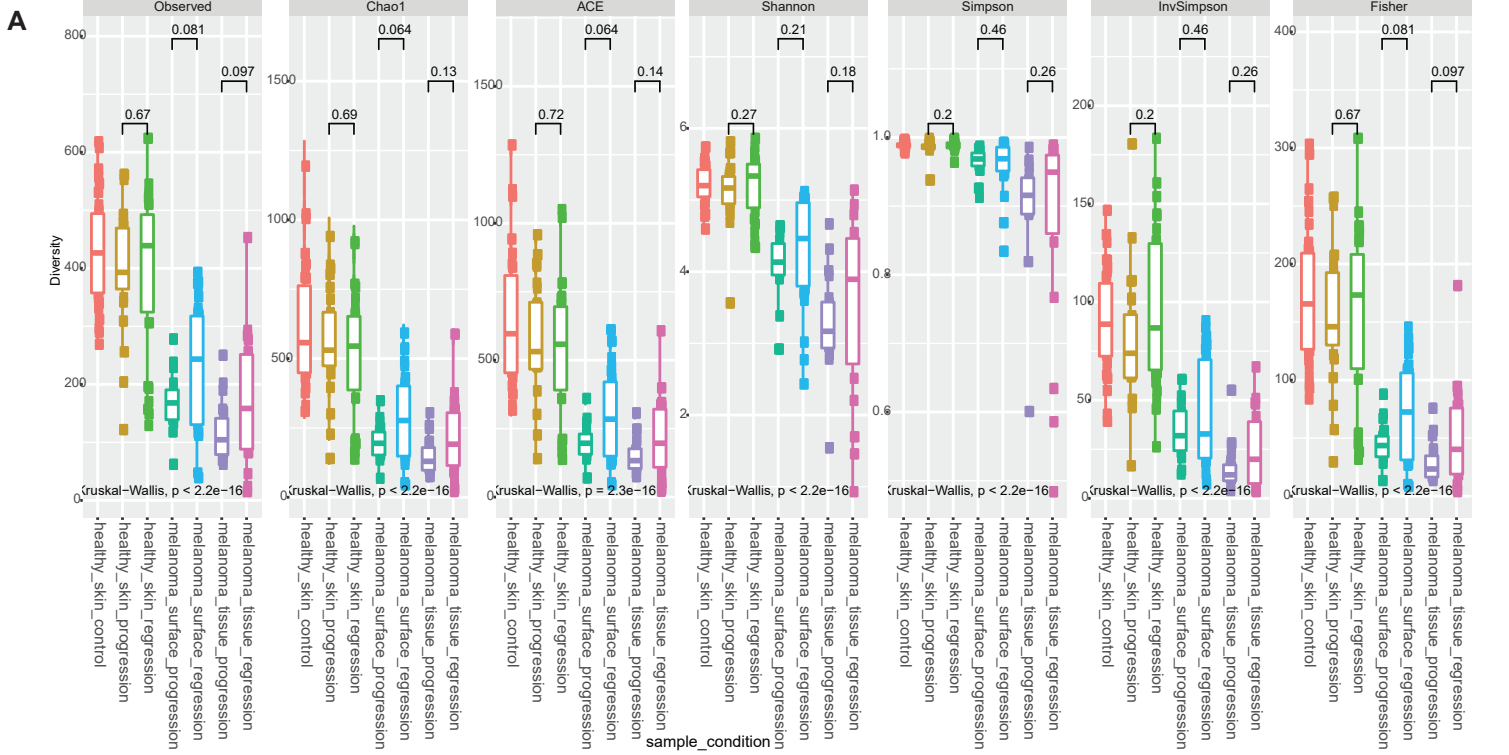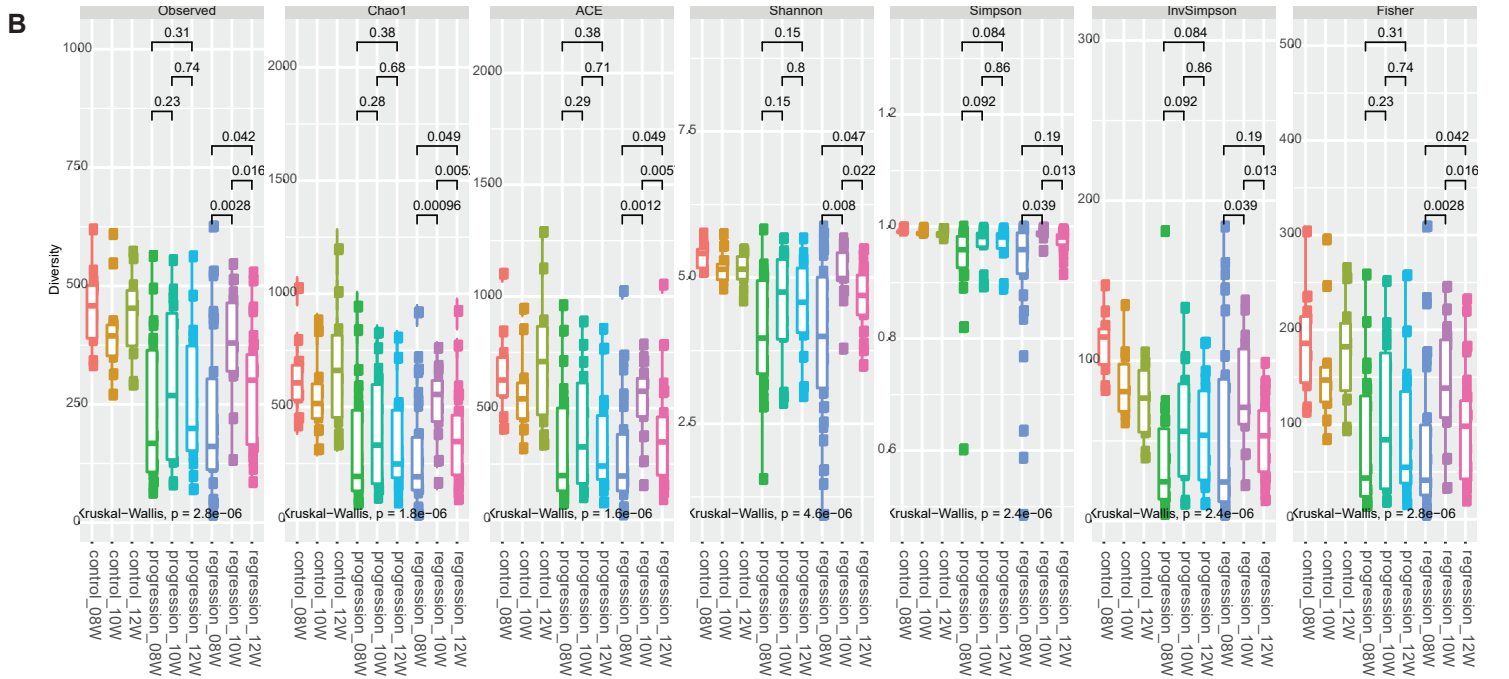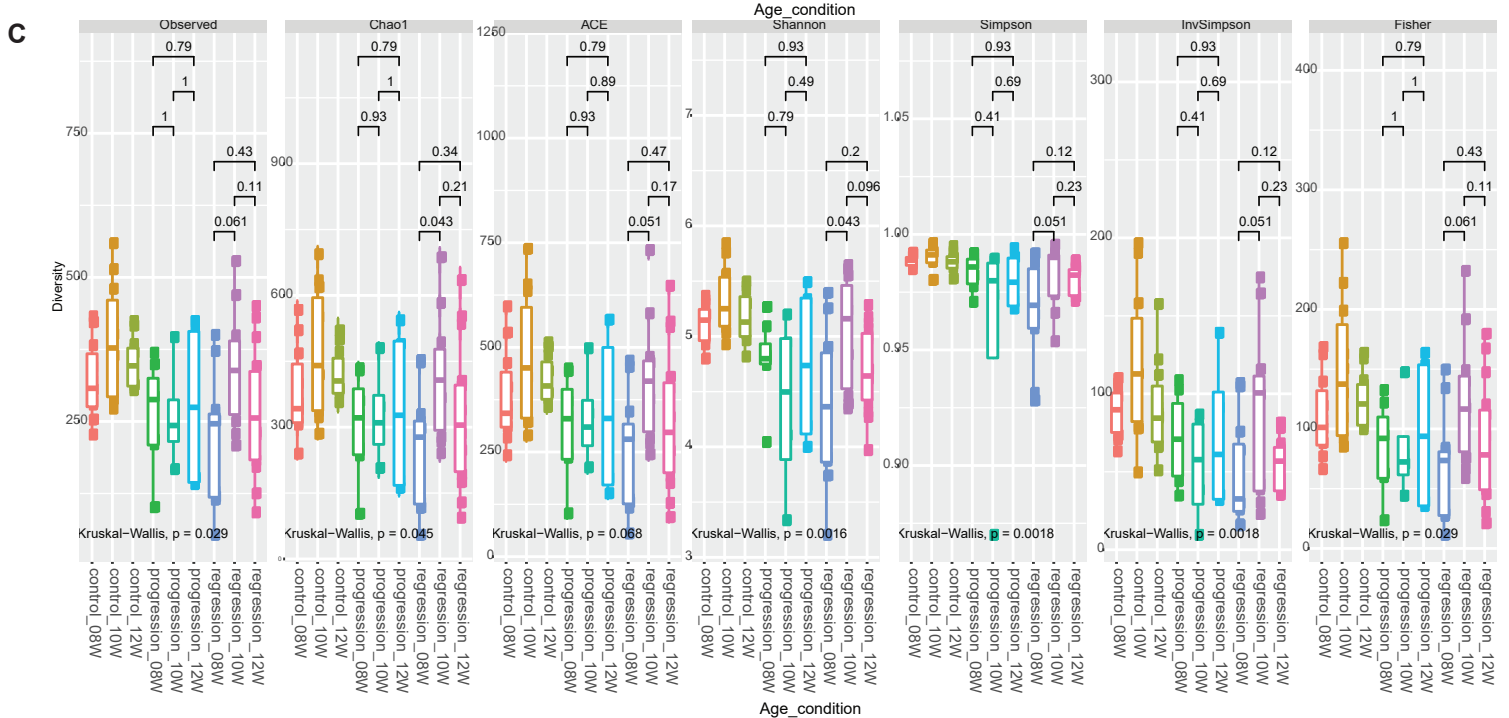

Supplement: Supplementary file 3 — Additional file 3. Comparison of the bacterialalpha diversity a) in the cutaneous microbiome of piglets with melanomaregression and piglets with melanoma progression in different cutaneous samples(healthy skin, melanoma surface and melanoma tissue), b) in the cutaneousmicrobiome of piglets with melanoma progression and melanoma regression atdifferent ages (8, 10, 12 weeks) and c) in faecal microbiome of piglets atdifferent stat (control, melanoma progression and melanoma regression)throughout the age (8, 10, 12 weeks). Bacterial diversity and richness wereestimated by different alpha diversity indexes: Observed species, Chao, Ace,Shannon, Simpson, Inverse Simpson and Fisher index. Kruskal–Wallis pairwisetest (p-value ≤ 0.05) was used to compare between different samples. [file 12866_2022_2458_MOESM3_ESM.pdf]

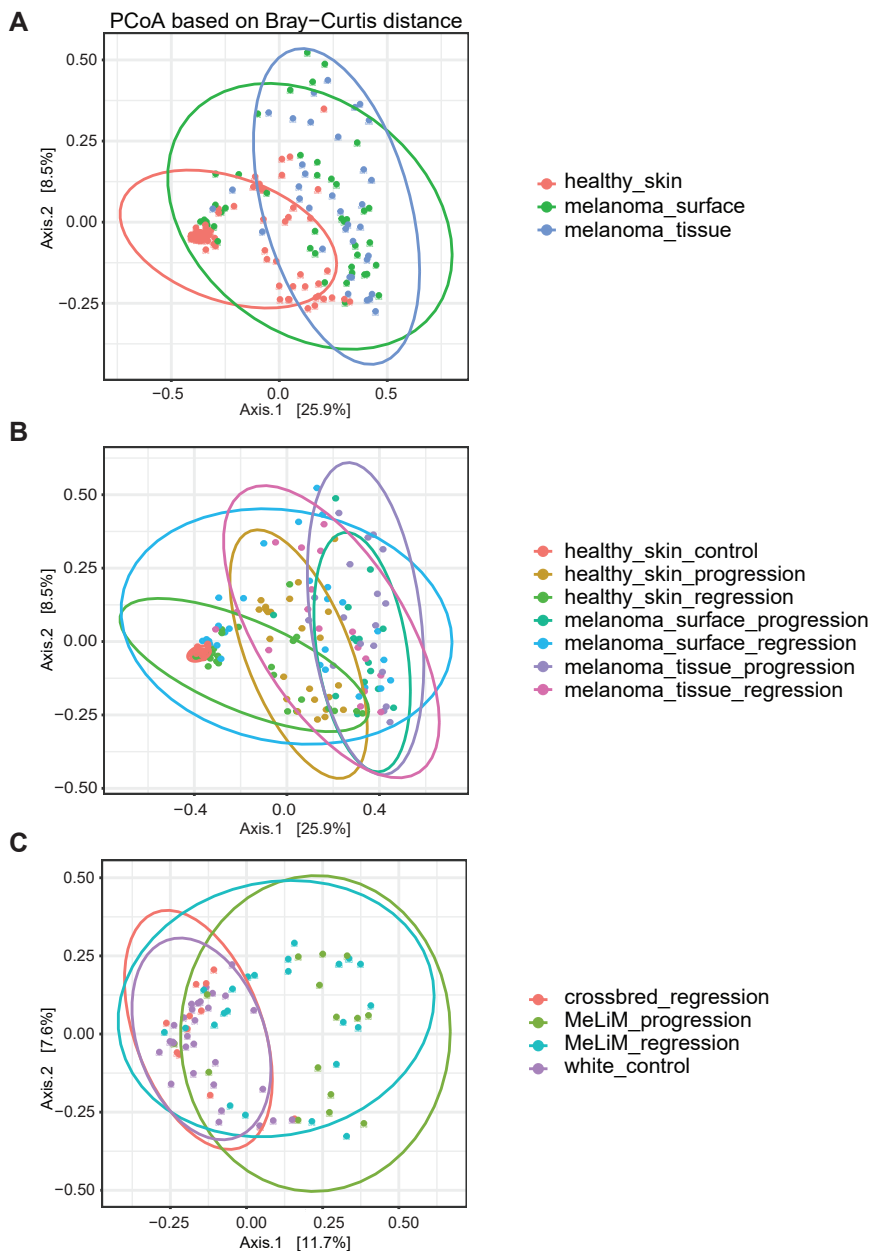

Supplement: Supplementary file 4 — Additional file 4. Beta diversity of bacterialcommunities using Principal Coordinate Analysis (PCoA) ordinations based on theBray Curtis distance matrix. The dissimilarities between bacterial communitieswere represented by regrouping in distinct clusters: a) in skin microbiomeamong different samples (healthy skin, melanoma surface and melanoma tissue, b)in skin microbiome among multiple samples in different disease conditions(control, melanoma progression and melanoma regression) and c) in faecalmicrobiome of different piglets groups (white control, crossbred with melanomaregression, MeLiM with melanoma regression and MeLiM with melanomaprogression). The confidence level of the ellipse was 95%. [file 12866_2022_2458_MOESM4_ESM.pdf]

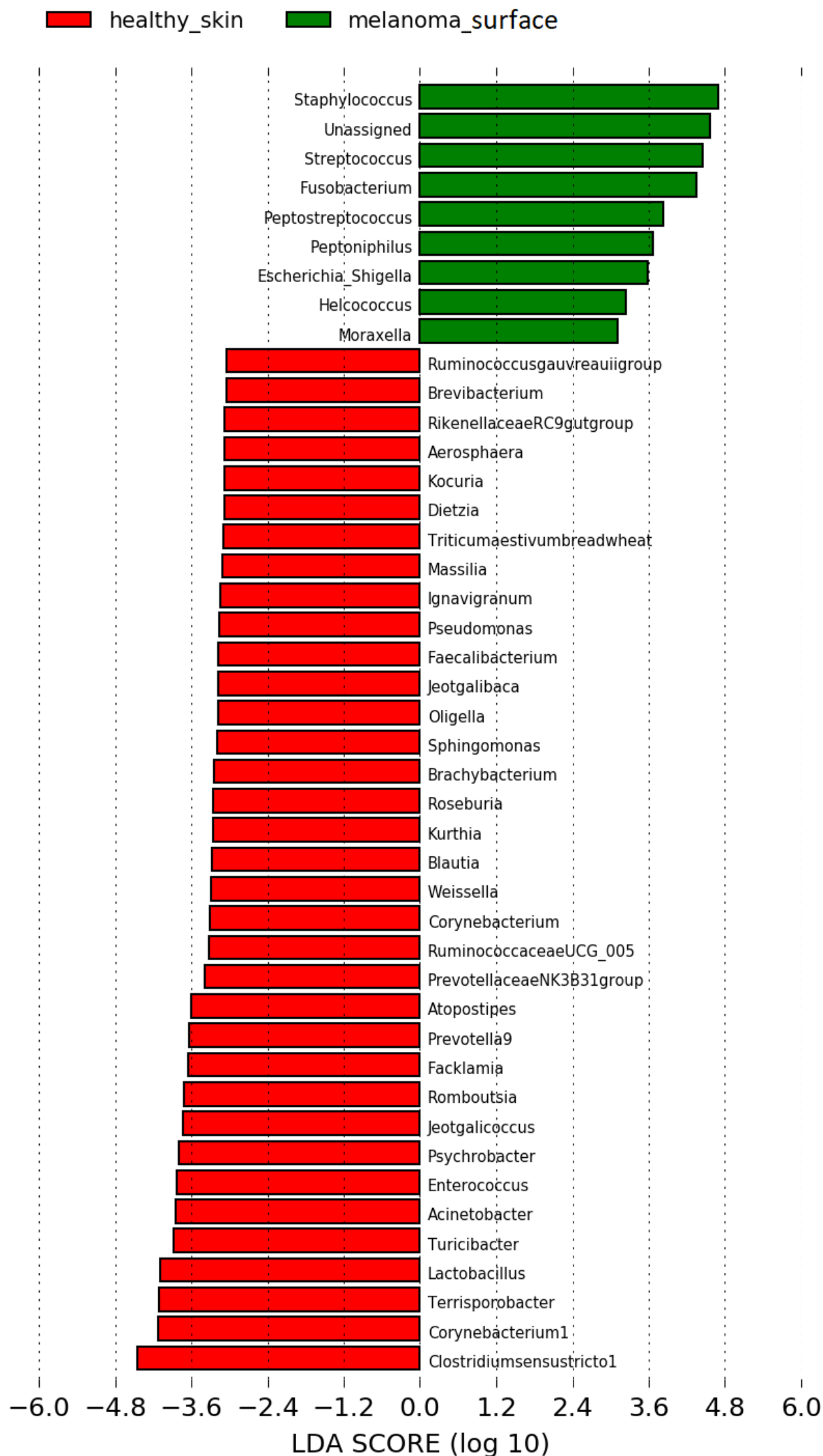

Supplement: Supplementary file 5 — Additional file 5. Linear discriminantanalysis (LDA) effect size (LEfSe) at genera level (i) in skin microbiomebetween: a) healthy skin and melanoma surface and b) healthy skin and melanomatissue, and (ii) in faecal microbiome c) between different conditions (healthycontrol, melanoma progression and melanoma regression) and d) between MeLiMpiglets and crossbred animals. Differential abundance between categories wasevaluated based on the factorial Kruskal-Wallis (KW) test and the pairwiseWilcoxon test (p < 0.05 and LDA score/effect-size threshold = 3). [file 12866_2022_2458_MOESM5_ESM.pdf]

healthy\_skin melanoma\_tissue

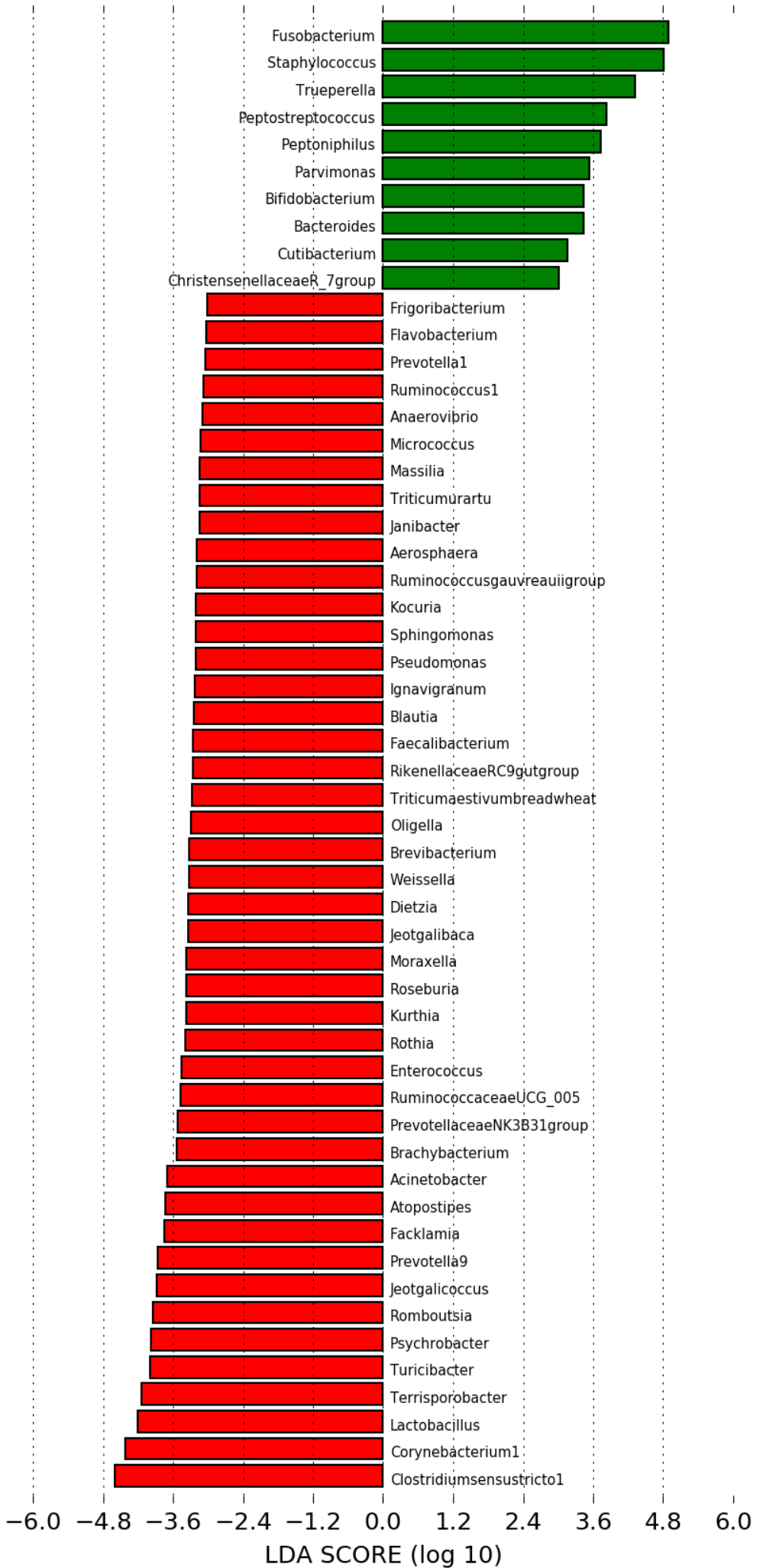

Supplement: Supplementary file 6 — Additional file 6. [file 12866_2022_2458_MOESM6_ESM.pdf]

control progression regression

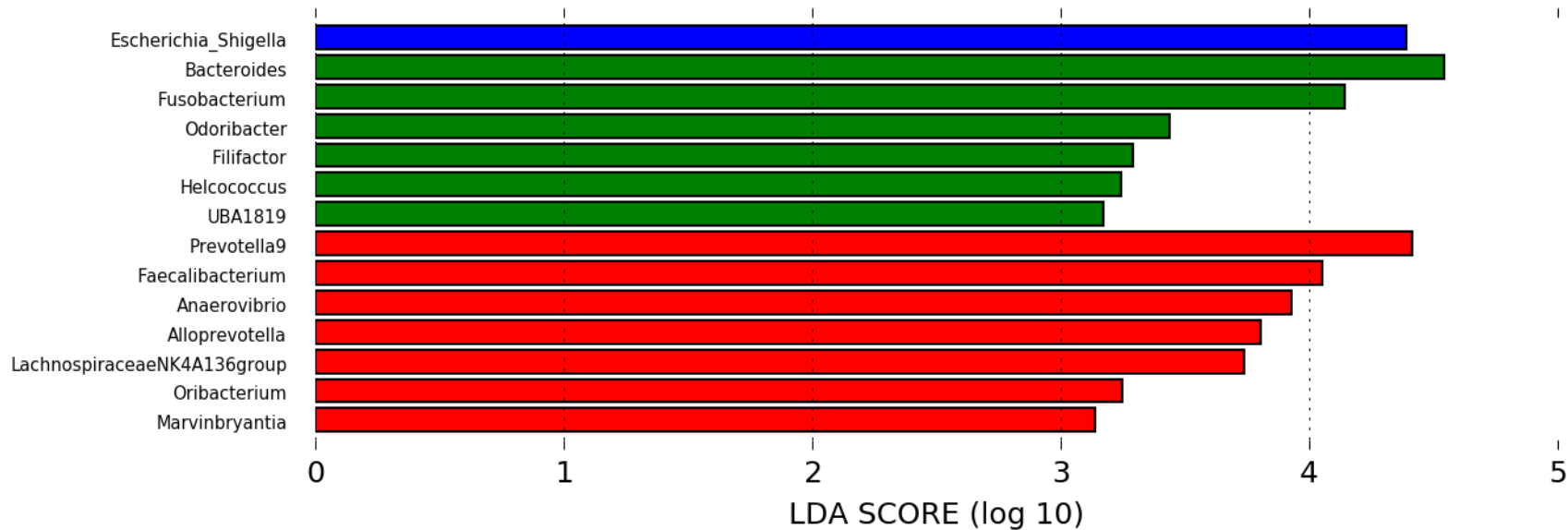

Supplement: Supplementary file 7 — Additional file 7. [file 12866_2022_2458_MOESM7_ESM.pdf]

MeLiM-stool      crossbred--stool

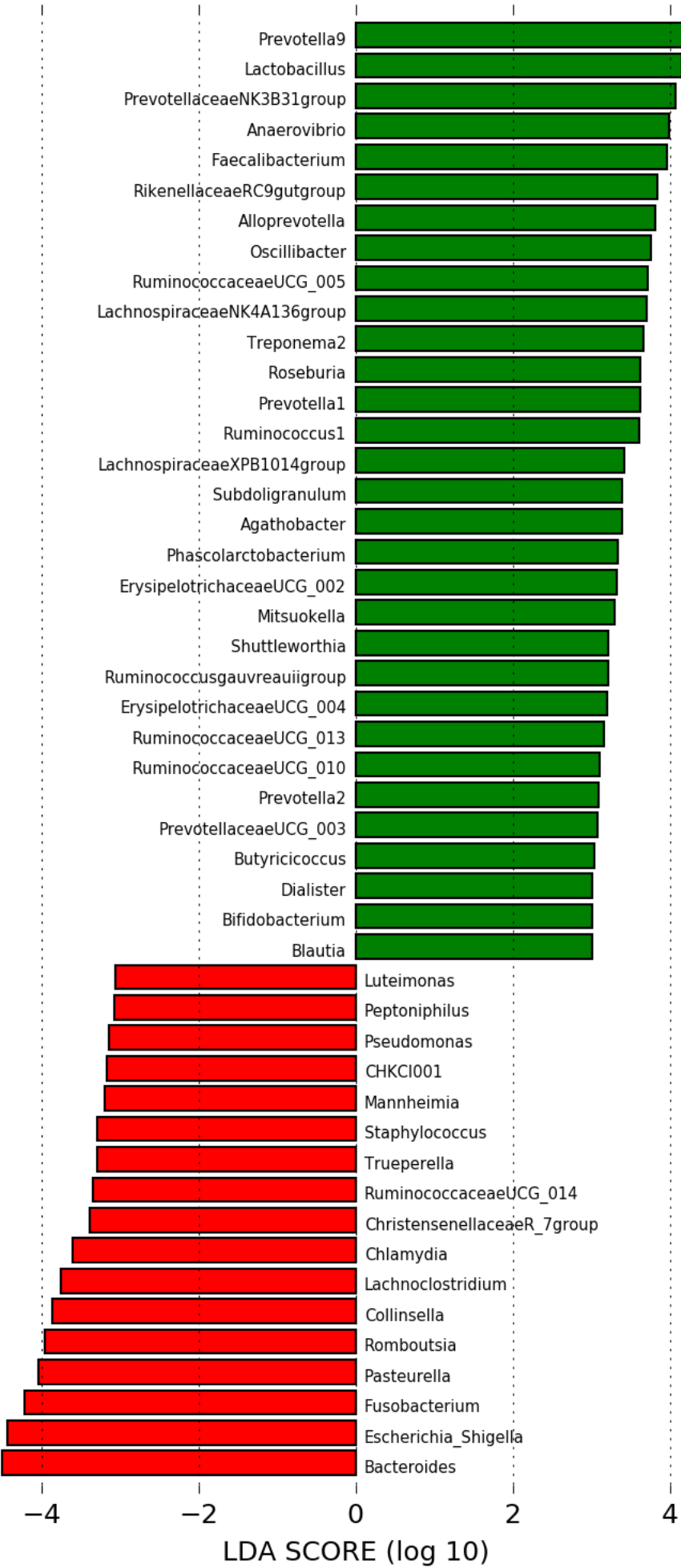

Supplement: Supplementary file 8 — Additional file 8. [file 12866_2022_2458_MOESM8_ESM.pdf]

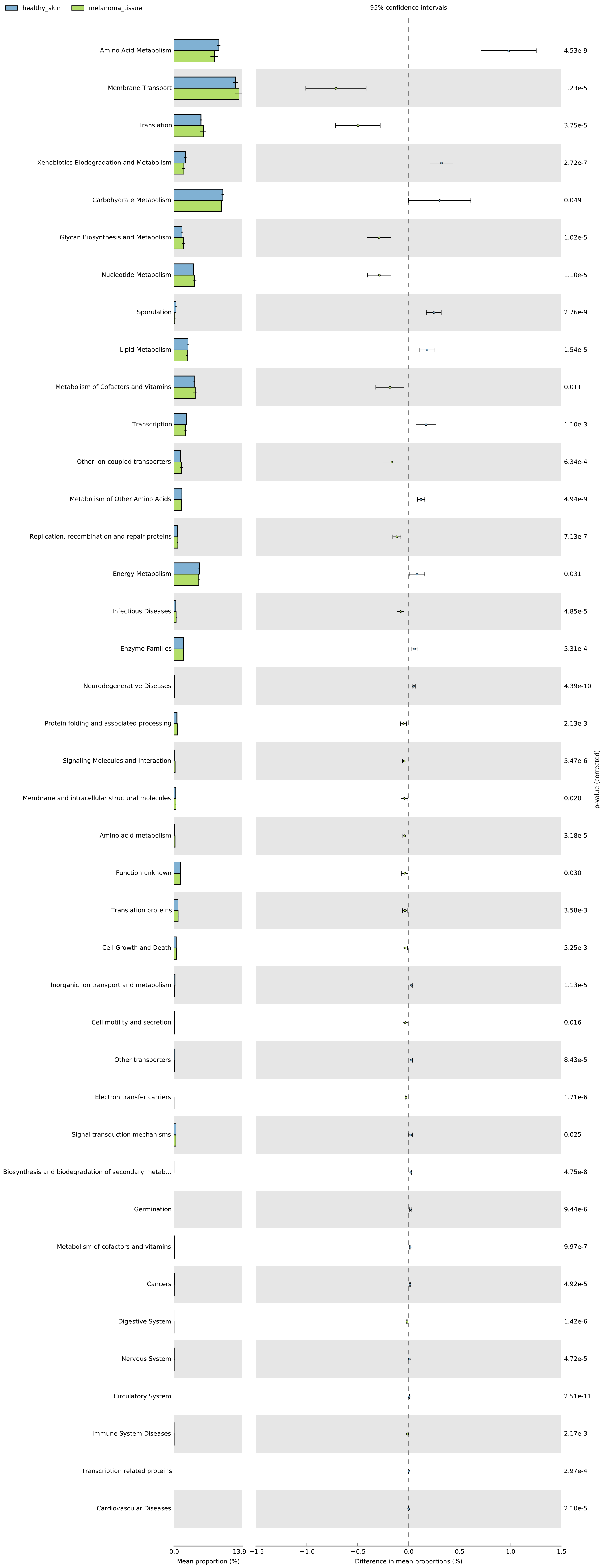

Supplement: Supplementary file 9 — Additional file 9. Functional pathway analysis ofthe cutaneous microbiome and faecal microbiome based on the KEGG database.Extended error bar plot identifying the significant differences in meanproportion (%) of predicted functional categories a) at second-level KEGGpathway between the healthy skin microbiome and melanoma tissue microbiome, b)at second-level KEGG pathway between melanoma progression and melanomaregression in melanoma tissue microbiome, c) at second-level KEGG pathwaybetween the faecal microbiome of MeLiM piglets and faecal microbiome ofcrossbred piglets and d) at third-level KEGG pathway between the faecalmicrobiome of MeLiM piglets with melanoma progression and MeLiM piglets withmelanoma regression using the STAMP software. The corrected p-values that wereshown on the right, were obtained from a Welch's t-test with the confidenceinterval (CI) method of Welch's inverted adjustment of 0.95 (p< 0.05). [file 12866_2022_2458_MOESM9_ESM.pdf]

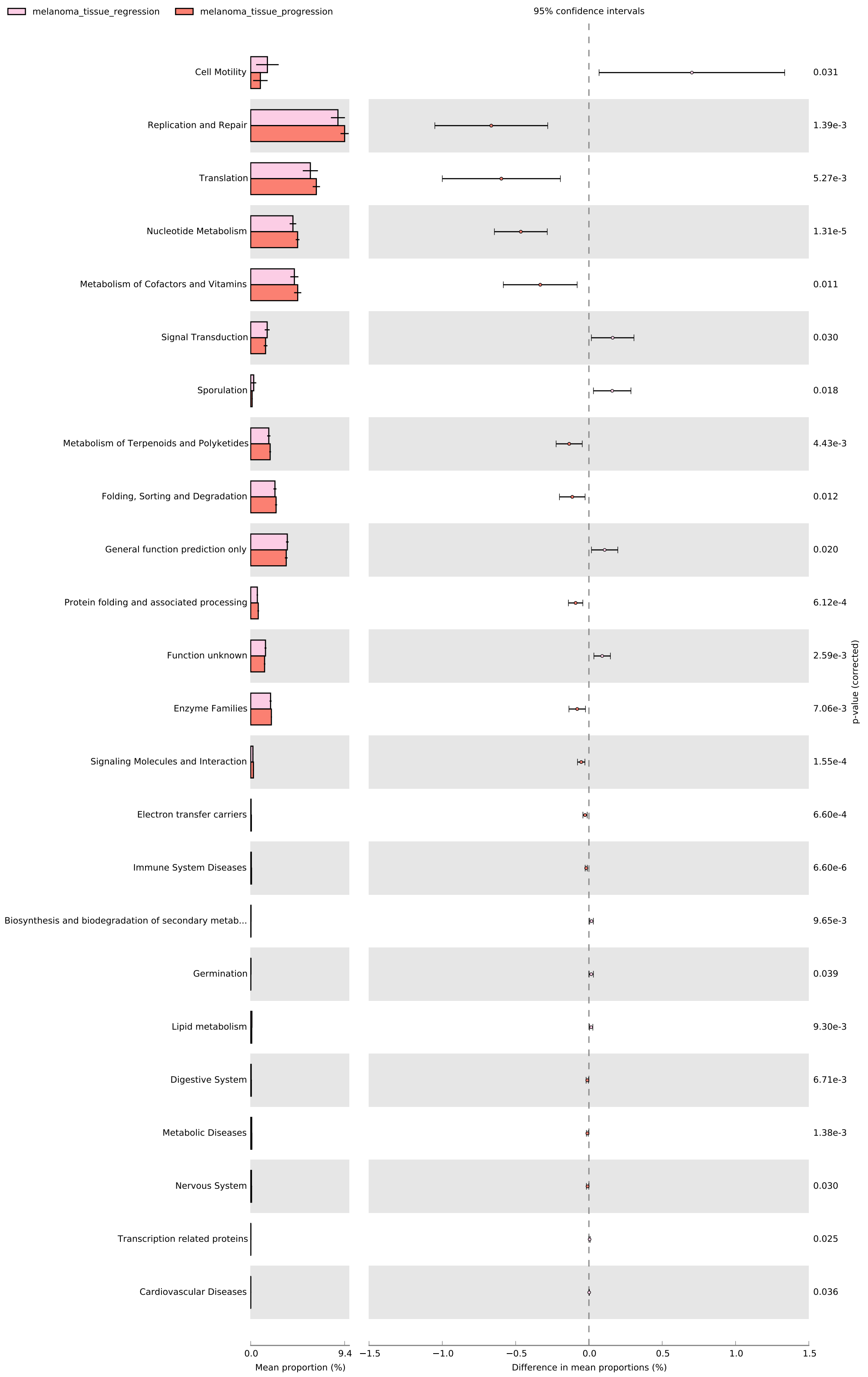

Supplement: Supplementary file 10 — Additional file 10. [file 12866_2022_2458_MOESM10_ESM.pdf]

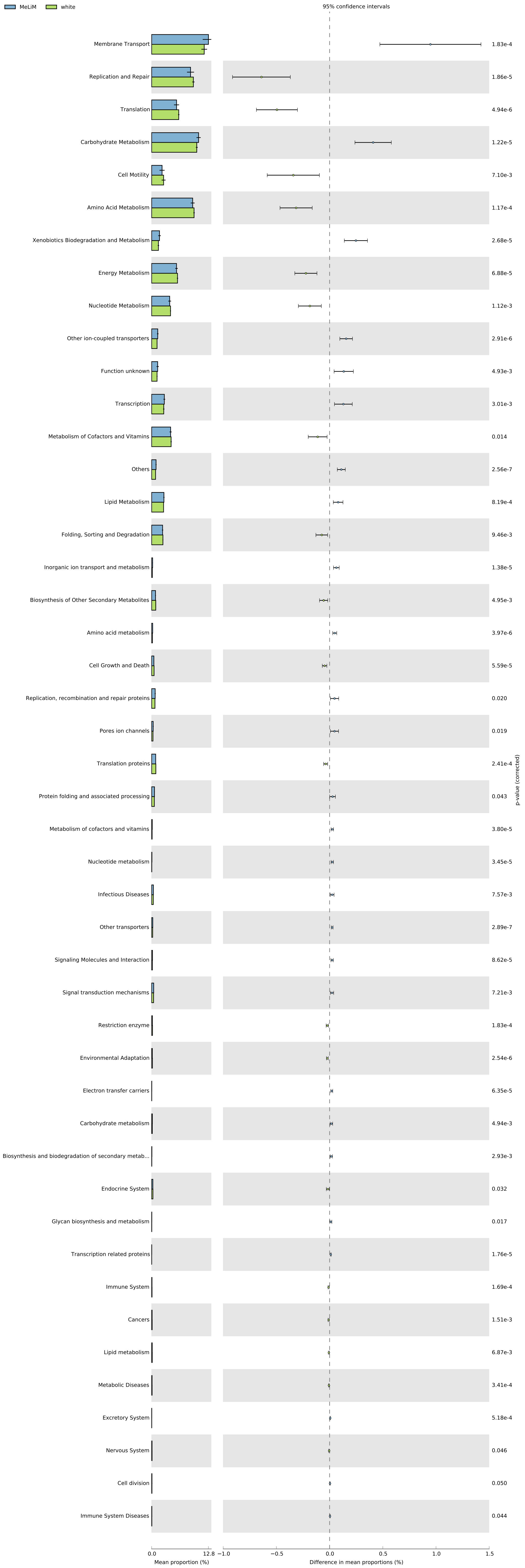

Supplement: Supplementary file 11 — Additional file 11. [file 12866_2022_2458_MOESM11_ESM.pdf]

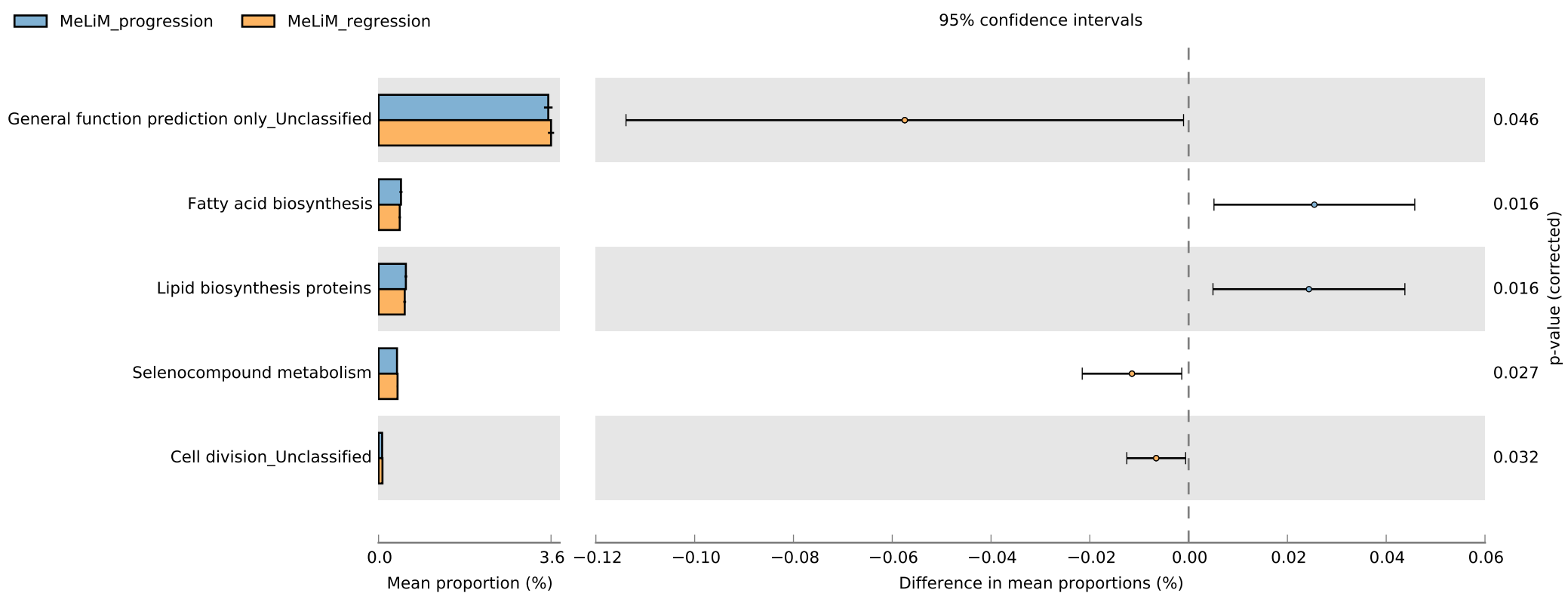

Supplement: Supplementary file 12 — Additional file 12. [file 12866_2022_2458_MOESM12_ESM.pdf]

healthy\_skin melanoma\_tissue

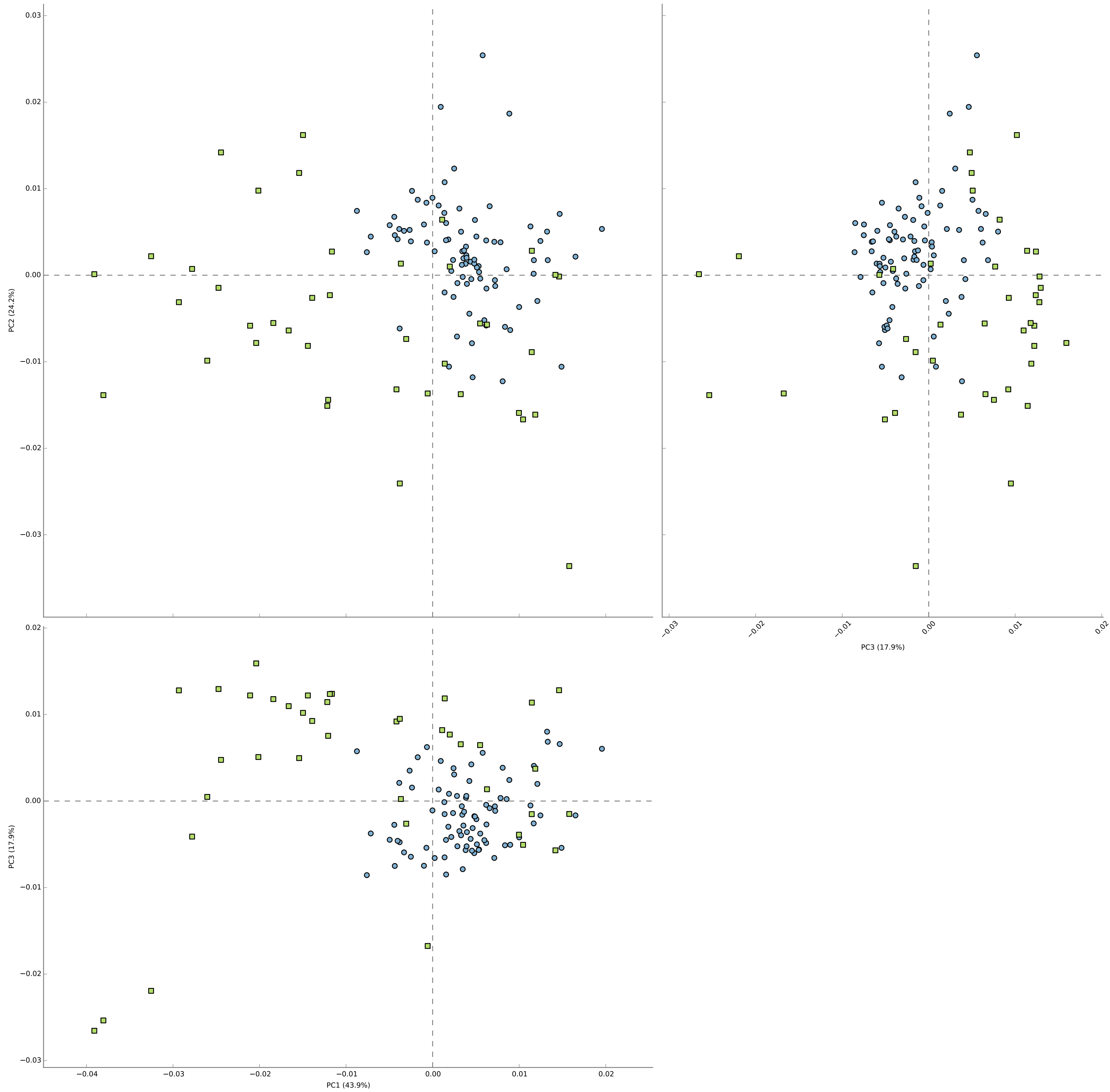

Supplement: Supplementary file 13 — Additional file 13. Principal ComponentAnalysis (PCA) of the predicted functional pathways at KEGG level 2 a) betweenthe healthy skin microbiome and melanoma tissue microbiome, b) between melanomaprogression and melanoma regression in melanoma tissue microbiome, c) betweenthe faecal microbiome of MeLiM piglets and faecal microbiome of crossbredpiglets and d) between the faecal microbiome of MeLiM piglets with melanomaprogression and MeLiM piglets with melanoma regression using the STAMP softwarebased on Non-corrected Welch'st-test type two-sided, with the confidenceinterval (CI)methodofWelch's invertedadjustment of 0.95(p< 0.05). [file 12866_2022_2458_MOESM13_ESM.pdf]

melanoma\_tissue\_regression melanoma\_tissue\_progression

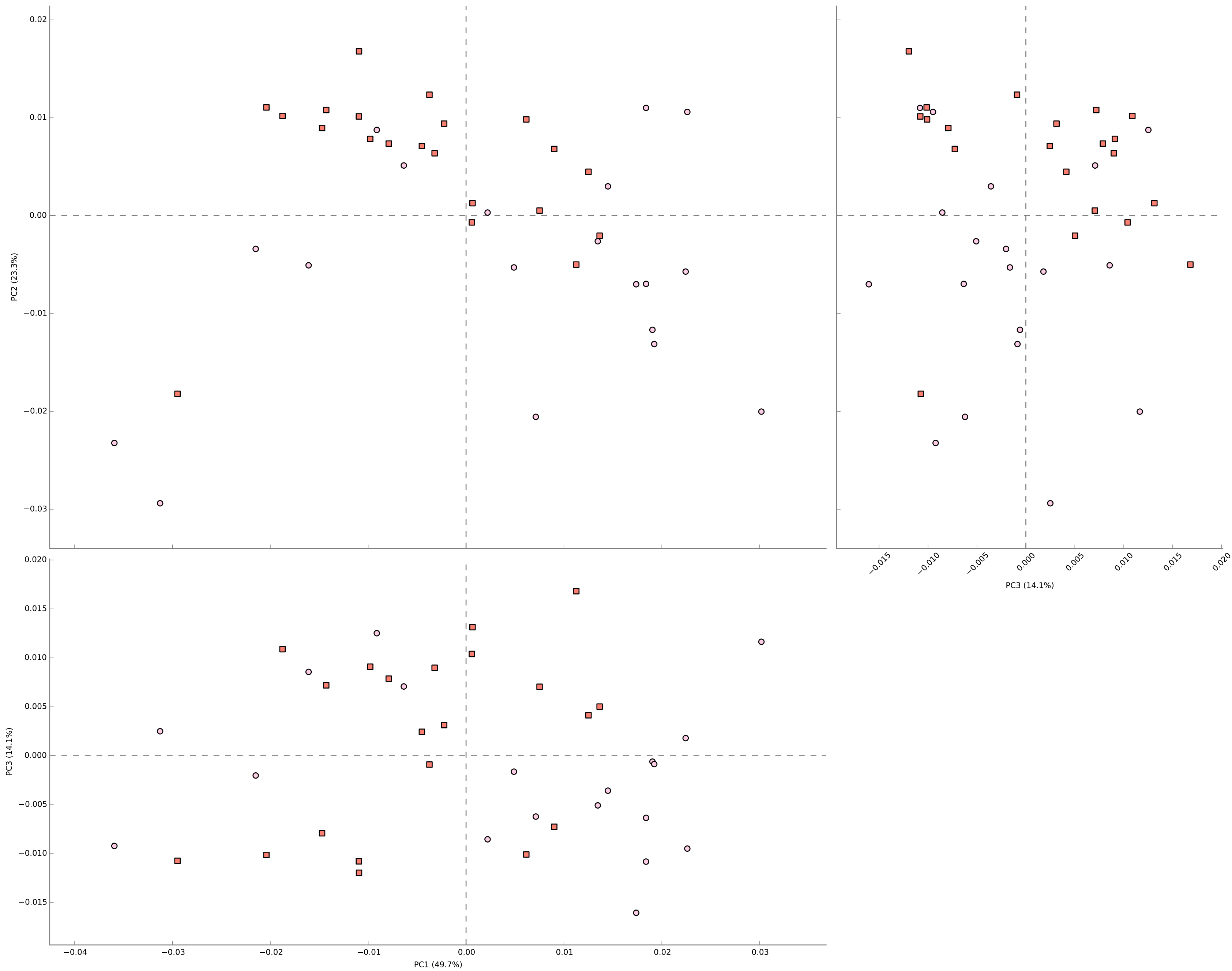

Supplement: Supplementary file 14 — Additional file 14. [file 12866_2022_2458_MOESM14_ESM.pdf]

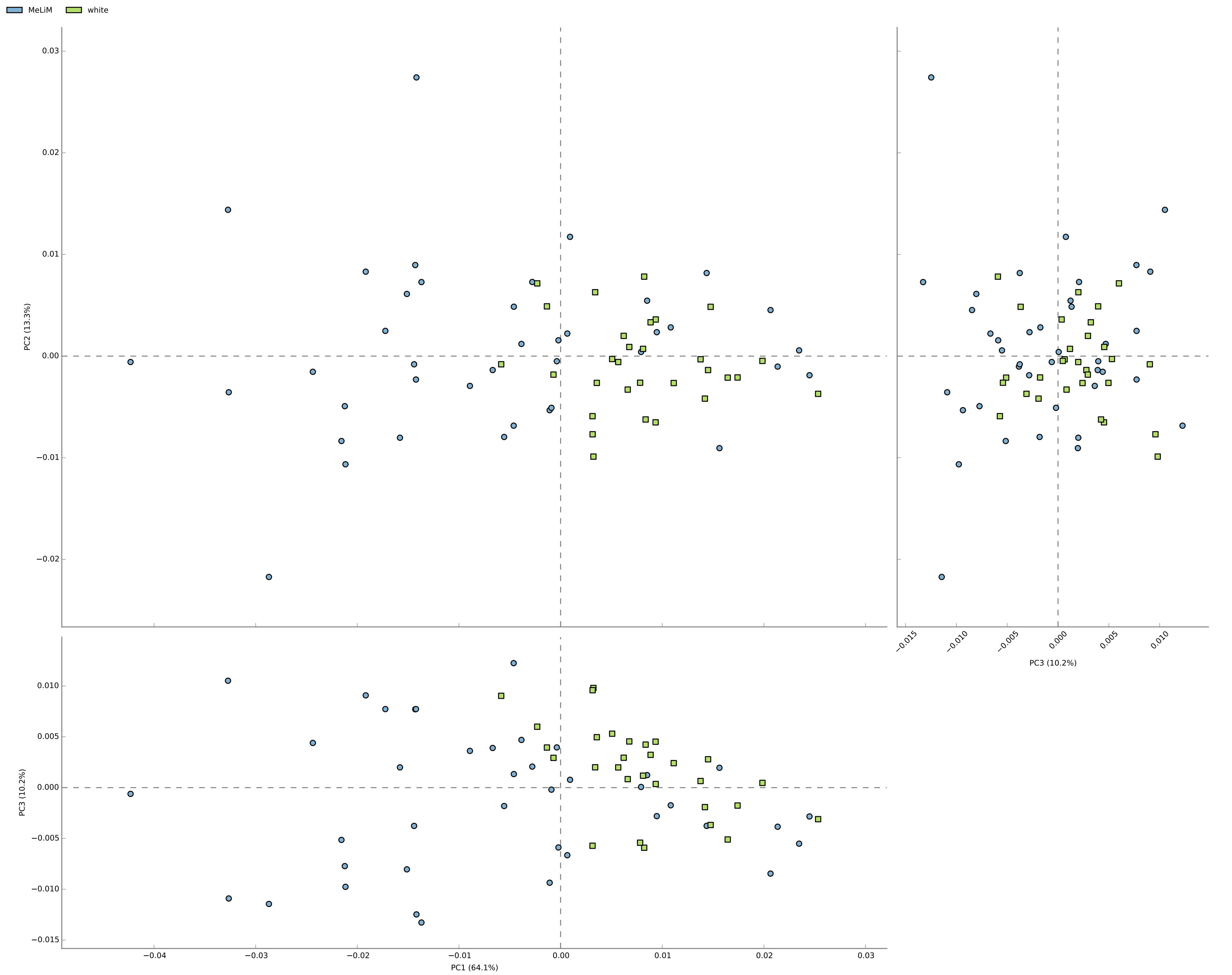

Supplement: Supplementary file 15 — Additional file 15. [file 12866_2022_2458_MOESM15_ESM.pdf]

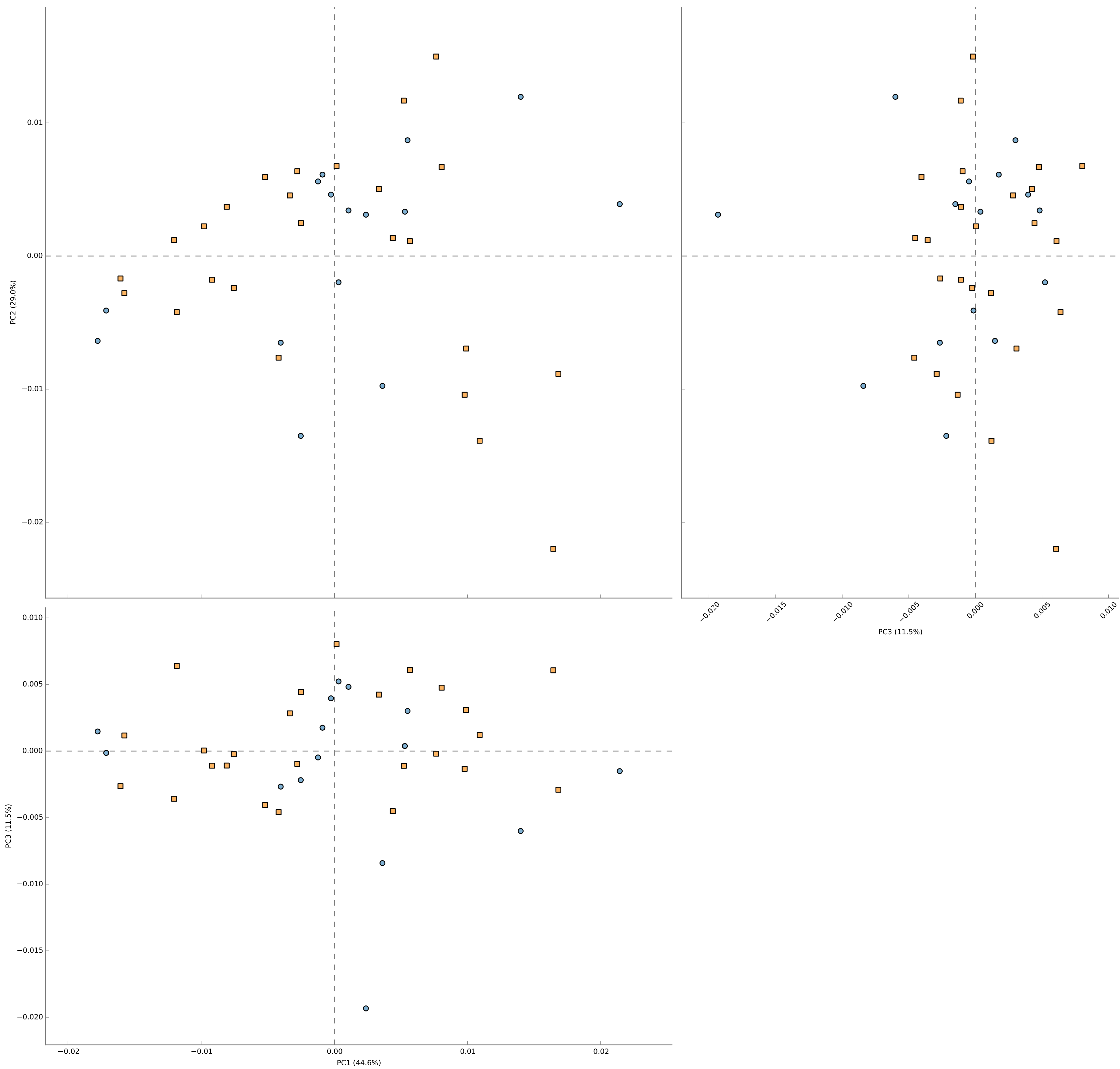

Supplement: Supplementary file 16 — Additional file 16. [file 12866_2022_2458_MOESM16_ESM.pdf]

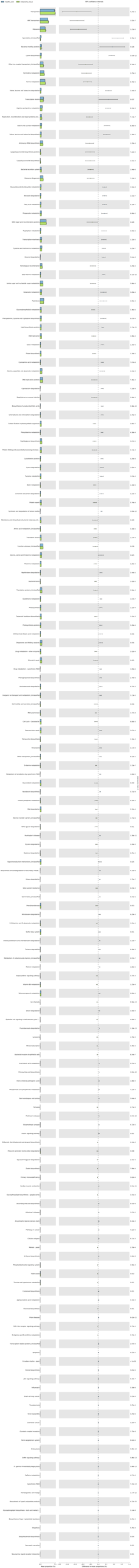

Supplement: Supplementary file 17 — Additional file 17. Functional pathway analysis of cutaneous microbiomebased on the KEGG database. Extended error bar plot identifying the significantdifferences in mean proportion (%) of predicted functional categories at level3 of KEGG pathway between the healthy skin microbiome and melanoma tissue microbiome. [file 12866_2022_2458_MOESM17_ESM.pdf]

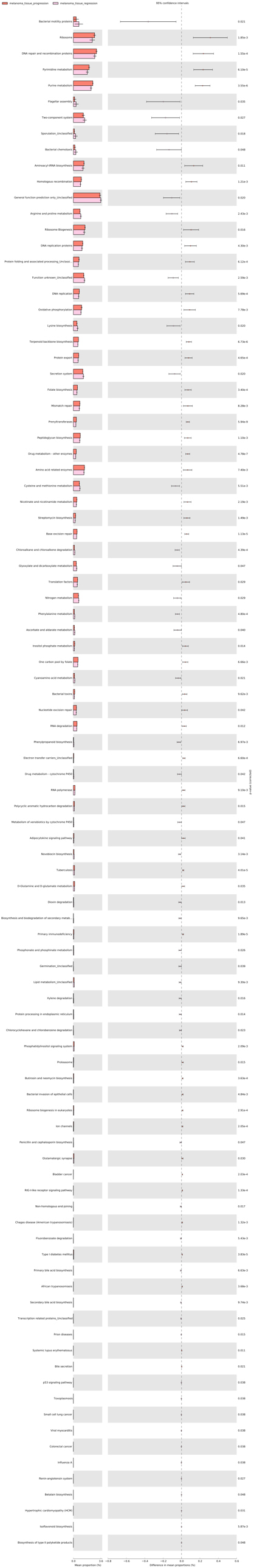

Supplement: Supplementary file 18 — Additional file 18. Functional pathway analysisof cutaneous microbiome based on the KEGG database. Extended error bar plotidentifying the significant differences in mean proportion (%) of predictedfunctional categories at level 3 of KEGG pathway between melanoma progressionand melanoma regression in melanoma tissue microbiome. [file 12866_2022_2458_MOESM18_ESM.pdf]

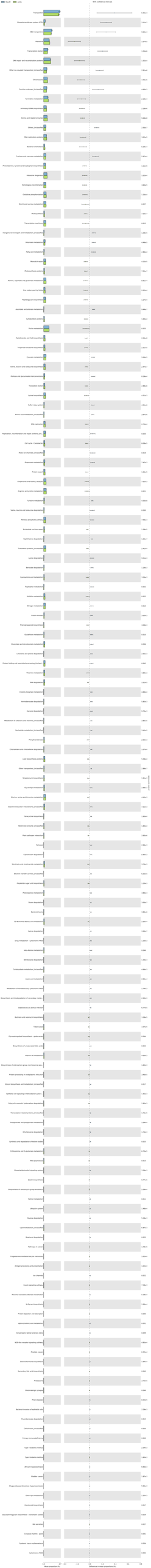

Supplement: Supplementary file 19 — Additional file 19. Functional pathway analysisof faecal microbiome based on the KEGG database. Extended error bar plotidentifying the significant differences in mean proportion (%) of predictedfunctional categories at level 3 of KEGG pathway between MeLiM piglets andcrossbred piglets. [file 12866_2022_2458_MOESM19_ESM.pdf]

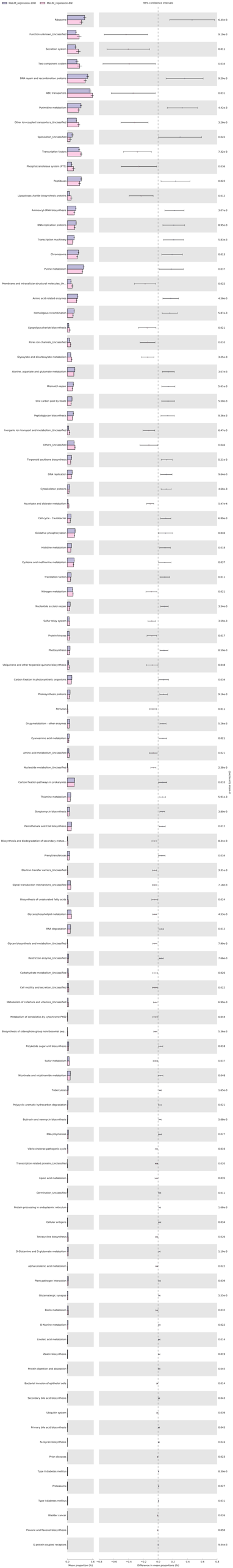

Supplement: Supplementary file 20 — Additional file 20. Functional pathway analysisof faecal microbiome based on the KEGG database. Extended error bar plotidentifying the significant differences in mean proportion (%) of predictedfunctional categories at second-level KEGG pathway between MeliM piglets withmelanoma regression at the age of 8 weeks and MeliM piglets with melanomaregression at the age of 10 weeks. [file 12866_2022_2458_MOESM20_ESM.pdf]

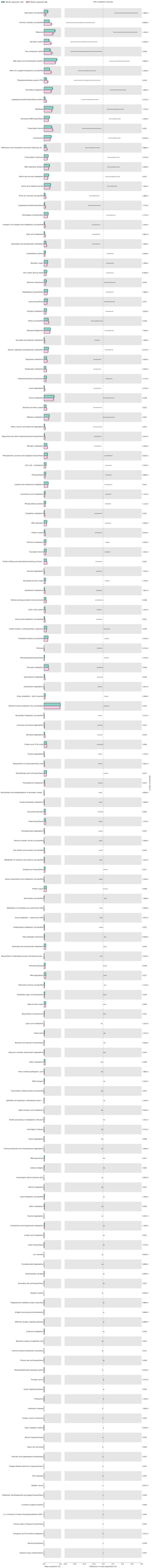

Supplement: Supplementary file 21 — Additional file 21. Functional pathway analysisof faecal microbiome based on the KEGG database. Extended error bar plotidentifying the significant differences in mean proportion (%) of predictedfunctional categories at second-level KEGG pathway between MeliM piglets withmelanoma regression at the age of 8 weeks and MeliM piglets with melanomaregression at the age of 12 weeks. [file 12866_2022_2458_MOESM21_ESM.pdf]
